# Diverse type 2 diabetes genetic risk factors functionally converge in a phenotype-focused gene network

**DOI:** 10.1371/journal.pcbi.1005816

**Published:** 2017-10-23

**Authors:** Cynthia Sandor, Nicola L. Beer, Caleb Webber

**Affiliations:** 1 Department of Physiology, Anatomy and Genetics, University of Oxford, Oxford, United Kingdom; 2 Oxford Centre for Diabetes, Endocrinology and Metabolism, Radcliffe Department of Medicine, University of Oxford, Oxford, United Kingdom; Mount Sinai School of Medicine, UNITED STATES

## Abstract

Type 2 Diabetes (T2D) constitutes a global health burden. Efforts to uncover predisposing genetic variation have been considerable, yet detailed knowledge of the underlying pathogenesis remains poor. Here, we constructed a T2D phenotypic-linkage network (T2D-PLN), by integrating diverse gene functional information that highlight genes, which when disrupted in mice, elicit similar T2D-relevant phenotypes. Sensitising the network to T2D-relevant phenotypes enabled significant functional convergence to be detected between genes implicated in monogenic or syndromic diabetes and genes lying within genomic regions associated with T2D common risk. We extended these analyses to a recent multiethnic T2D case-control exome of 12,940 individuals that found no evidence of T2D risk association for rare frequency variants outside of previously known T2D risk loci. Examining associations involving protein-truncating variants (PTV), most at low population frequencies, the T2D-PLN was able to identify a convergent set of biological pathways that were perturbed within four of five independent T2D case/control ethnic sets of 2000 to 5000 exomes each. These same pathways were found to be over-represented among both known monogenic or syndromic diabetes genes and genes within T2D-associated common risk loci. Our study demonstrates convergent biology amongst variants representing different classes of T2D genetic risk. Although convergence was observed at the pathway level, few of the contributing genes were found in common between different cohorts or variant classes, most notably between the exome variant sets which suggests that future rare variant studies may be better focusing their power onto a single population of recent common ancestry.

## Author summary

Type 2 Diabetes (T2D) has been the subject of a large number of genetic studies that have identified multiple and heterogeneous risk variants. However, our knowledge of the underlying perturbed molecular pathways influenced by these variants remains poor. Exploiting our prior knowledge of T2D-relevant mammalian phenotypes, we developed a novel network-based approach focused on bringing sources of information together about gene function that are most relevant to T2D. This approach is able to identify convergent biology amongst variants representing different classes of T2D genetic risk, including low-effect common variants, high-effect rare variants, and variants drawn from recent population-based exome studies. Our study shows that a broad range of genetic variation influences T2D-risk through a limited number of biological pathways.

## Introduction

Type 2 Diabetes (T2D) affects over 285 million people worldwide and constitutes 90% of all adult diabetes [1]. Over the last decade, there has been substantial investment in efforts to identify genetic variants predisposing towards T2D. The major successes have involved genome-wide association (GWA) studies that have identified over 80 common variant signals influencing T2D-risk [2–5]. With advances in sequencing technology, it has become possible to evaluate the contribution of low frequency and rare variants to T2D-risk, though to date, these studies remain relatively underpowered [6–8]. As with many other common diseases, the variants so far identified explain only a small proportion of disease risk variance [4].

Most of the variants implicated by common variant GWAS map to regulatory sequence: there has been only limited progress at identifying the protein-coding genes through which these exercise their effects. This has inhibited efforts to characterize the underlying gene networks and molecular processes that are perturbed in individuals predisposed to T2D-risk. However, the integration of diverse types of genomic data gathered from T2D-relevant tissues [9] combined with a growing inventory of coding variants causally linked to T2D-risk is now providing a body of information that can be harnessed to highlight the most promising positional candidates at GWAS loci. In turn, these data provide a substrate for network-based methods that, on the assumption that disease-associated genes operate through a limited number of shared molecular processes, seek to detect patterns of functional convergence across sets of associated genes and regions.

There are a variety of such methods available. These include gene-set enrichment methods, which consider whether a set of candidate disease genes is over-represented for a particular functional annotation [10] and gene-network methods, which identify functional associations as the clustering of genes of interest within a larger network of genes whose juxtapositions are defined by information such as protein-protein interactions (PPI) [11] or co-expression [12]. A major limitation of these approaches derives from the functional datasets employed: the functional annotations of many genes are incomplete, and no single dataset can provide a complete picture of the functional relationships between genes.

To address this, we recently proposed a method that combines information from multiple data types to identify functional similarities between genes. Our approach exploits phenotypic information from the over 7000 genes whose function has been experimentally perturbed in the mouse [13, 14] and evaluates the ability of different functional data types to predict whether or not knockout of the orthologues of a given pair of human genes will yield similar phenotypes. By weighting those data types accordingly, we can integrate them to generate a single combined measure of functional similarity between gene pairs. The resulting network of pairwise gene functional similarities is termed a phenotypic-linkage network (PLN). Clustering variant genes from exome studies, we showed that this has a greater specificity and sensitivity than analyses that use a single data type and comparable methods that integrate multiple data types [13].

The PLN seeks to detect convergent functionality between genes focusing on the likelihood that groups of genes influence the same phenotype irrespective of etiology, as opposed to other methods that look to infer the causal relationships between genes, for example through inferring gene regulatory networks [15], [16] [17].

Functional gene networks have been previously employed to identify of candidate genes from GWAS or other genetic/genomic studies [21]. Prioritisation methods, such as GeneMANIA, Endeavour [22, 23], require of a list of known genes associated with the disorder in question whose functional commonalities are identified by the method in order to prioritise other genes exhibiting similar functionality. This is limiting as currently known disease genes may only represent a subset of etiologies and thus prevent the identification of genes involved in novel disease etiologies. More recent approaches have sought to exploit functional genomics information particular to tissue/cell types likely relevant to a given disease through tissue-specific genome-wide functional interaction networks based on the hypothesis that the relevant disease genes act in a specific cell/tissue type [18]. However this approach requires knowledge of the disease cell type(s) which remain unclear for many disorders (e.g. Migraine [19]). Furthermore, many cell types might contribute to the same disease with pathways crossing cell types, for example insulin secretion by pancreatic islet beta cells and insulin response in liver, skeletal muscle, adipose tissue are all involved in the pathophysiology of T2D [20].

Here we propose an approach to focus functional genomics data towards a particular disorder based on the following hypothesis: while our generalized PLN approach makes use of the full range of mouse model phenotypes, most disease studies are focused towards identifying genes that influence a subset of phenotypes that are relevant to the disease in question and thus are most interested in those types of functional genomics data most informative for those particular phenotypes. Accordingly, we developed a novel T2D-specific PLN that integrates only those data types providing an accurate prediction as to whether two genes are likely to have a similar influence on T2D-relevant mouse phenotypes. By focusing in this way, we downweight uninformative data that may both contribute irrelevant functional associations and obscure associations of interest but remain uncommitted to a particular tissue-specific etiology. Initially, we compared the abilities of a general PLN and the T2D-PLN to detection functional relationships between known or suspected T2D-risk genes. To address this, we defined subnetworks within each PLN, termed gene communities, and tested for the enrichment of particular communities using three different types of T2D genetic risk loci: (a) genes containing rare coding alleles considered causal for multiple forms of monogenic and syndromic diabetes; (b) genes mapping to T2D risk intervals, defined at both genome-wide and sub-genome-wide significance, identified through common variant GWAS; and (c) genes containing low-frequency and rare protein-truncating variant alleles from five distinct ethnic samples that show the most promising evidence of association to T2D in exome sequencing data. Uniquely, the phenotype-focused T2D gene network is able to identify significant and convergent functionality within all T2D-risk candidate gene sets, both individually and when combined, illustrating that convergent pathways are disrupted by T2D genetic variants of different frequencies and effect sizes.

## Results

### Constructing a disease-specific phenotypic-linkage gene network

**Fig 1:**
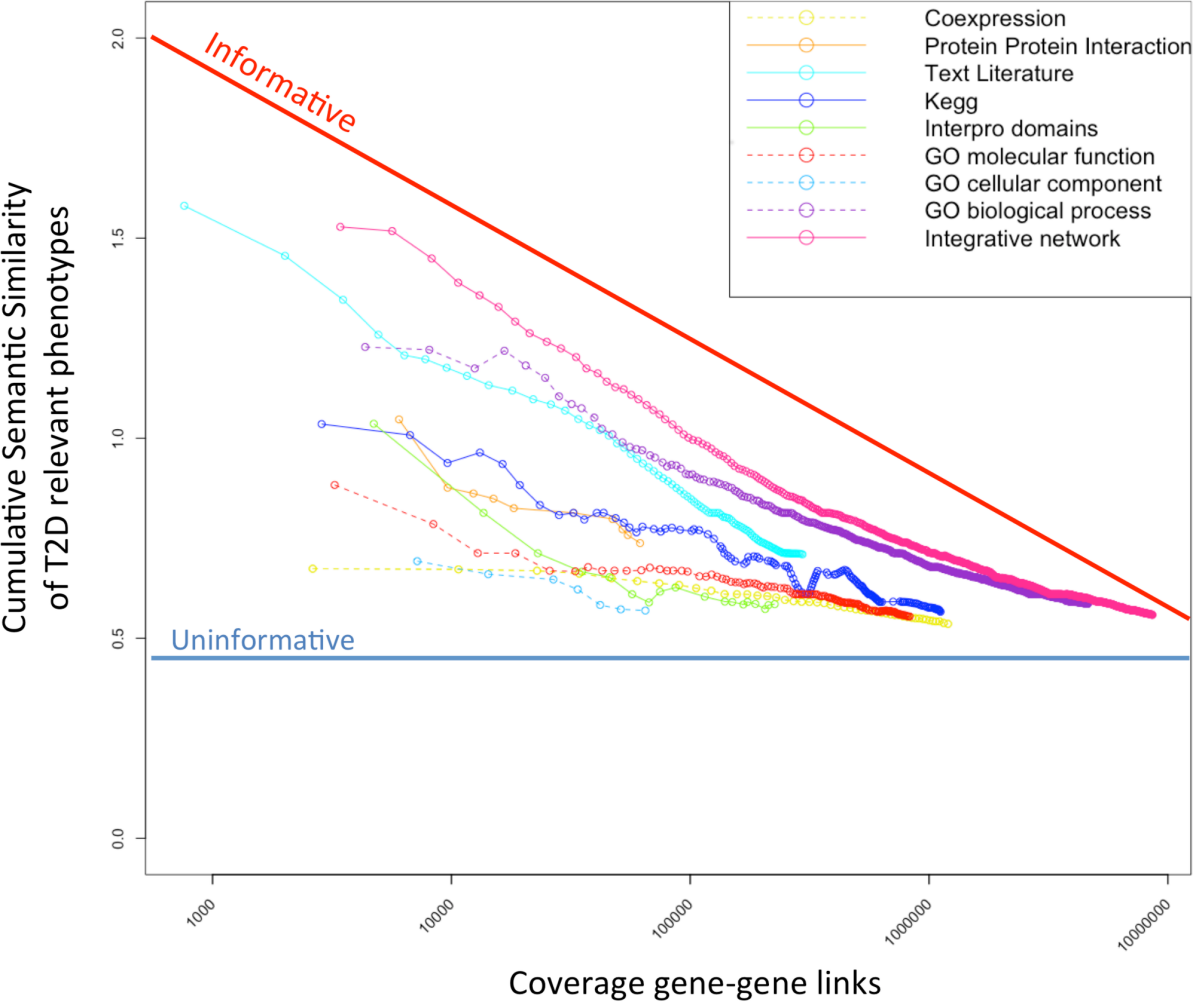
Type 2 diabetes phenotypic linkage network (T2D-PLN) construction. The T2D-PLN was constructed by evaluating the abilities of different data types to predict the similarity in the T2D-relevant phenotypes following the determined disruption of pairs of genes in the mouse[13]. Different data types provide information of characteristic accuracy over different sets of genes. For each of the data types, we have ordered the gene pairs by their scores, divided them to bins of 1000 pairs and calculated the median cumulative semantic similarity between gene pairs’ mouse knockout phenotypes (Y-axis) at different levels of coverage (X-axis). The pink curve represents the final T2D-PLN. The Y-axis gives the semantic similarity of the phenotypes from the pairwise mouse model comparisons, while the X-axis gives the number of gene-gene links covered. (TIF 1.3 Mb).

Our approach is based on a method developed by Honti *et al*. [13], which combines multiple sources of information about gene function (e.g. RNA expression, protein-protein interactions, functional annotations and more) into a single measure that reflects the likelihood that any given pair of genes influence the same mammalian phenotype (**Fig 1**). The key step in this approach is to evaluate, for pairs of genes annotated with the same information type, how well each distinct source of information is able to predict the similarity of phenotypes observed following disruption of those genes’ unique orthologues in the mouse. From this evaluation, for each gene pair in turn, each available data type is accorded a score: these scores are then combined, across all data sources, into a single weighted “link” (**Fig 1**). The links between all gene pairs define the phenotypic linkage network (PLN) (**Fig 1**).

In this study, we refine this general approach by restricting those evaluations of functional data types to consider only the prediction of mouse phenotypes likely to be relevant to T2D (**Fig 1**). To achieve this, we identified 38 T2D-relevant Mouse Phenotype Ontology (MPO) terms (**S1 Table**). These mapped into 3 of the 33 over-arching MPO phenotypic categories: endocrine/exocrine phenotypes (MP:0005379), homeostasis/metabolism phenotypes (MP:0005376) and adipose tissue phenotypes (MP:0005375). We generated a type 2 diabetes phenotypic linkage network (T2D-PLN) by considering functional data types according to their ability to infer whether disruption of pairs of murine orthologues leads to phenotypic similarity for traits within only these three MPO categories. The resulting T2D-PLN comprised 7,811,440 weighted direct links between 20529 protein coding genes, (**Fig 1**; **S1 Fig**; **S2 Table** shows the contribution of each data type). Note that both the T2D-PLN and the general PLN integrate exactly the same functional genomics data but weight these data differently.

### The T2D-PLN outperforms a general PLN in detecting functional links between two types of diabetes risk genetic factors

**Fig 2:**
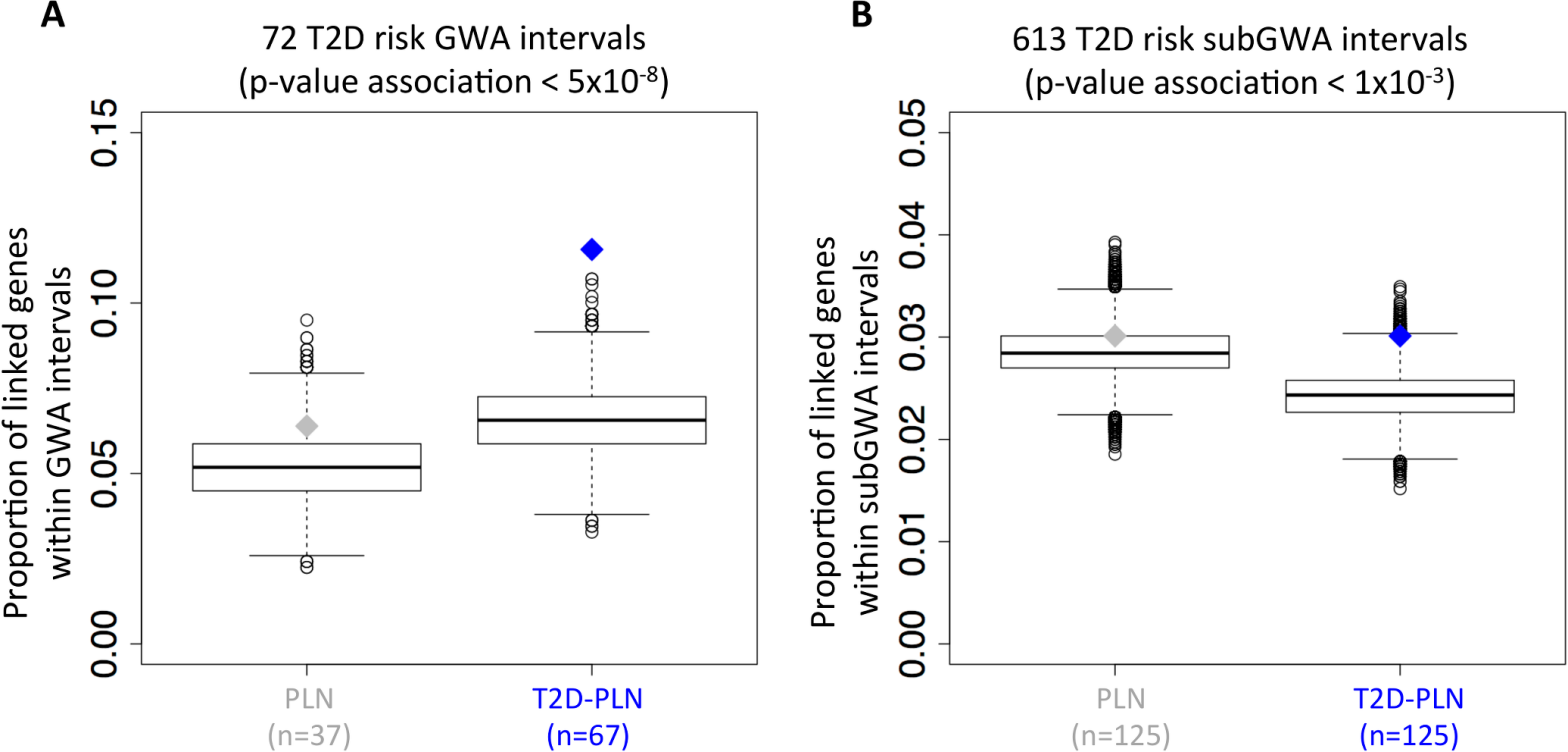
T2D-PLN versus PLN performance. The T2D-PLN outperforms the PLN in detecting functional links between 32 monogenic and syndromic diabetes genes (Mono-Syn) and **(A)** genes lying within 72 known T2D-risk GWAS intervals and **(B)** 613 new T2D-risk GWAS intervals defined by considering SNPs with a p-value < 10^−3^ and after excluding the 72 known GWAS intervals. The gray and blue boxplots represent the random distribution of number of linked genes within GWAS intervals for general PLN and T2D-PLN respectively. The red point represents the observed number of linked genes within GWAS interval. (TIF 475 kb).

To illustrate the gain of sensitivity and specificity resulting from this novel PLN, we compared the abilities of a generic PLN and the T2D-PLN to detect functional relationships between a well-defined set of genes implicated in diabetes pathogenesis and two sets likely to be enriched for such genes. The “Mono-Syn” set includes 32 genes that contain rare coding alleles of large effect considered causal for a number of monogenic and syndromic forms of diabetes including maturity onset diabetes of the young and neonatal diabetes reported in Online Mendelian Inheritance in Man database (OMIM) (http://www.omim.org/) (**S3 Table**). The “GWAS” set comprises a total of 579 protein-coding genes that map to 72 loci implicated in T2D predisposition through common variant GWAS. These 72 loci were selected from the 82 established T2D GWAS loci (as of mid-2014), after excluding the 10 intervals that harbour genes contained in the “Mono-Syn” set (**S4 Table**). Lastly, the “subGWAS” set comprises 4150 genes gathered from 613 loci defined by lead SNPs that showed lesser degrees of common variant association to T2D. Specifically, this was the set of 613 independent SNPs attaining an association p-value<10^−3^ in a recently-completed GWAS meta-analysis of mostly European individuals[4] (http://diagram-consortium.org) but excluding the 72 T2D GWAS intervals already considered and any intervals encompassing “Mono-Syn” genes. Notably, the functional annotations of the “subGWAS” genes are unlikely to be biased by studies examining their relevance to diabetes. At each “GWAS” and “subGWAS” locus, a combination of linkage disequilibrium decline and physical distance from the most strongly associated variant had been used to define plausible locus intervals. In contrast to members of the “Mono-Syn” set, only a minority of the 579 genes in the GWAS set (~10–15%) are likely to be directly relevant to mechanisms of T2D predisposition and even fewer in the subGWAS set. We considered all genes in the “GWAS” and “subGWAS” sets as potential candidate genes and followed a diffusion approach whereby associations were allowed to “flow out” within the network from the 32 “Mono-Syn” genes to surrounding genes and beyond according to the strength of the functional links between them. Considering all genes within the T2D-PLN with significant links to Mono-Syn genes, we found that the “GWAS” and “subGWAS” gene sets contained an excess of genes functionally-associated to the Mono-Syn gene set (67 T2D GWAS interval genes, *p* = 2x10^-5^; 125 “subGWAS” genes; T2D-PLN *p* = 5x10^-3)^ (**Fig 2**and **S2 Fig**). No equivalent signal was seen with the generic PLN (37 T2D “GWAS” genes, *p* = 0.11; 125 T2D “subGWAS” genes, *p* = 0.26) (**Fig 2**and **S2 Fig**). Compared to our standard PLN, the T2D-PLN was more sensitive and specific in identifying functional associations between 32 “Mono-Syn” genes and genes in each of the “GWAS” and “subGWAS” gene sets showing that evaluating functional genomics data on T2D-relevant mammalian phenotypes significantly increases the sensitivity to detect convergent functionality between these gene sets.

### Different classes of T2D genetic risk variants functionally converge within T2D-PLN

A limitation of the network diffusion approach employed above is that it relies on predefining a set of genes known to be causally implicated in diabetes—such as the 32 “Mono-Syn” genes. This has two unwelcome consequences. First, it may lead to uneven representation of the etiological pathways implicated in T2D pathogenesis, and limit the opportunities to detect genes involving novel mechanisms. Secondly, these index genes are, by their very nature, likely to be have been particularly well-studied, with extensive functional connections and the potential for ascertainment bias[24].

To enable a more hypothesis-free approach, we fragmented the T2D-PLN gene network into a series of “communities”, each containing clusters of densely-interconnected genes, retaining 18 communities formed from at least 20 genes (**Methods**). We looked for evidence of a disproportionate aggregation of eight sets of known and candidate T2D-risk genes covering a spectrum of T2D-association confidence, risk, population frequency and bias including: three previously described gene sets (**(i)** “Mono-Syn”, **(ii)** “GWAS” and **(iii)** “subGWAS”)) and **(iv-viii)** five other gene sets from recently-acquired exome-sequencing data from the T2D-GENES and GoT2D consortia which examined 12,940 participants (approximately equal numbers of cases and controls) from five major ethnic groups (European, South Asian, East Asian, Hispanic and African-American) and found no evidence of T2D risk association for rare frequency variants [8]. Focusing exclusively on those variants predicted to lead to protein-truncation (PTVs) defined by Fuchsberger *et al*. [8], we selected the 300 most strongly T2D-associated genes for each of the 5 ethnic-specific case-control sample sets (SKAT-O test[25]).

We found evidence of disproportionate aggregation of gene set members across these communities, particularly within Community 5, where “Mono-Syn” genes (13/32 genes, corrected *p* = 5x10^-3^) (**S5 Table**), “subGWAS” genes (267/613 GWAS loci, corrected *p* = 0.046) (**S5 Table**) and PTV-associated genes for both Hispanic and South Asian samples were significantly enriched (FDR < 0.05; **S6 Table**), while nominal enrichments of “GWAS” genes (**S5 Table)** and PTV-associated genes for both East Asian and African-American samples were also observed (*p* < 0.05; **S6 Table**). Our findings retained significance for the PTV-associated gene sets when we varied the number of most-associated genes per ethnic sample considered (**S7 Table**).

**Fig 3:**
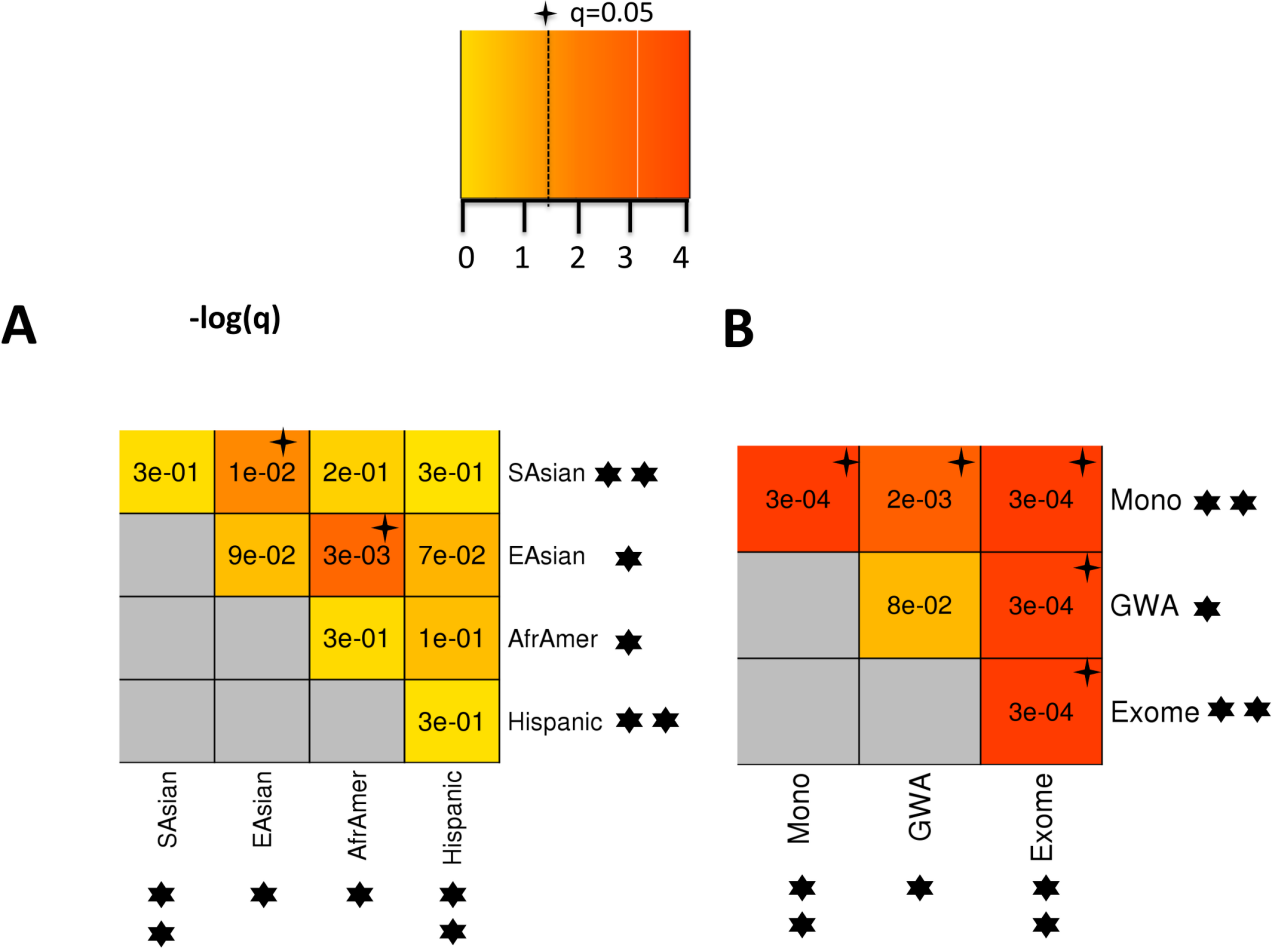
Clustering analyses of different T2D-risk candidate gene sets within Community 5. **(A)** Clustering analyses between different genes set impacted by PT-variants within Community 5. We considered in four different cohorts (South Asian, East Asian, African-American and Hispanic) the 300 most associated genes based on SKAT-O test p-values and that belonged to Community 5. **(B)** Clustering analyses of gene sets associated with different T2D genetics risk factors: (1) monogenic and syndromic diabetes genes (Mono) (2) genes harbored by 72 reference T2D GWAS intervals (GWAS) (3) genes impacted by PT-variants in four different population cohorts (genes considered in A but taken together). Empirical p-values were obtained by comparing the sum of link weights between genes within the two gene-sets combined as compared to randomly sampled gene sets from the Community 5 matched in number, coding length and gene connectivity (degree). Four-point stars denote significant functional clustering between respective variant sets (FDR-corrected). 6-point stars denote individual variant sets that were found to be enriched with Community 5 members, 2 stars denoting FDR-corrected significance and 1 star denoting nominal significance. (TIF 523 kb).

**Fig 4:**
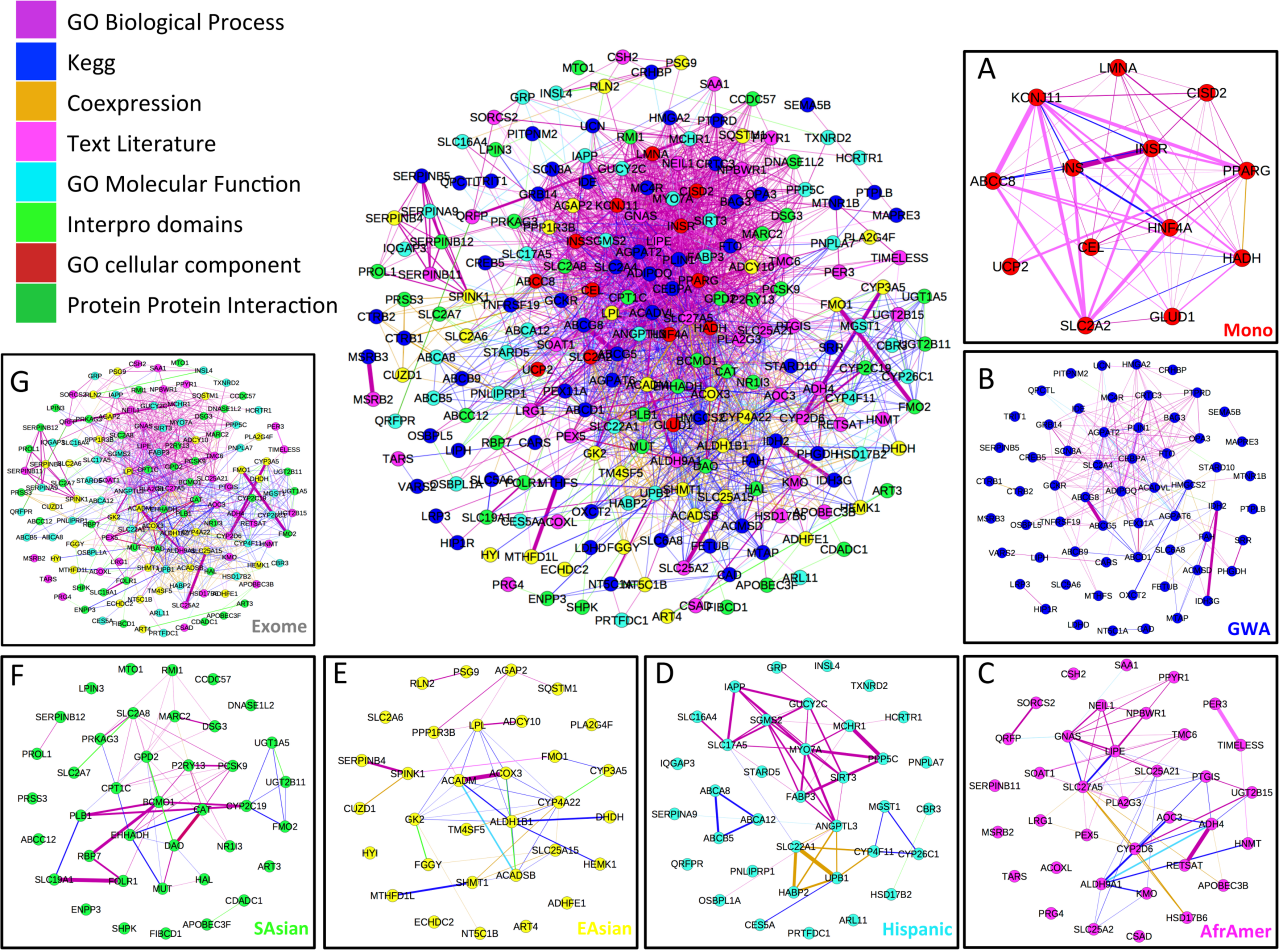
Sets of known and candidate T2D-risk genes and their functional associations within the T2D-PLN Community 5. Each named dot represents a known (panel **A**) or candidate (panels **B-G**) T2D-risk genes. Panel **A**: 13 monogenic and syndromic (Mono). Panel **B**: 71 genes residing within 72 T2D-risk GWAS intervals. Panels C-G: 40, 42, 51, 45 and 168 genes impacted by T2D-risk PT-variants in the African-American, Hispanic, East-Asian, South-Asian samples and then all samples except the [non-significant] European sample respectively. The conserved juxtapositions of the subnetworks contributed by each of the sets of known and candidate genes (panels **A-F**) to the combined network (panel **H**) are shown in **S4 Fig**. The colour of the link connecting two genes indicates the strongest information source supporting the functional association. (TIF 6.7 Mb).

Although there was little overlap in the Community 5 gene members that contributed to the signals in the four ethnic exome substudies (**S3 Fig**), likely resulting from ethnic-specific variants, we found that PTV-associated genes from the East Asian, South Asian and African-American exomes converged on a significant sub-cluster within Community 5 as compared to randomly sampled Community 5 genes (**S8 Table**) *(p* = 3x10^-4^) (**Fig 3B**). Furthermore, when we expanded these analyses to consider also the “Mono-Syn” and “GWAS” and all genes impacted by PT-variants in four different population cohorts (“Exome”) contributing to the Community 5 enrichment signals described earlier, we again found non-random aggregation of signal within that Community (**Fig 3B**). These findings are consistent with a model whereby diverse approaches to risk allele detection highlight associations, which converge towards a common T2D etiopathology (**Fig 3B**; **Fig 4; S4 Fig**; **S9 Table**).

To better understand the lack of overlap in the sets of Community 5 PT-variant genes identified for the four ethnic samples, for each set of genes, we performed a burden test for T2D for those genes in the other 3 ethnic samples (**S10 Table, S5 Fig**). We found no evidence of a burden in any other ethnic sample except that sample in which these genes were identified, suggesting that while different PT-variants impact the same genes within the same population, they affect different genes involved in the same pathways in different population. These results suggest population specific risks for genes. However, a significant SKAT-O test may also arise from protective, as well as deleterious, variants within a gene. To determine the risk/protective directionality of PTVs, we performed logistic regression analyses to predict the case/control status of each individual within each of the ethnic-specific samples as a function of the number of Community 5 PTV-associated genes showing enrichment. We found an enrichment in risk PTVs (positive odds ratio) in African-American and South Asian sample (which was significant p = 1.51x10-2 OR = 1.19 (95% confidence interval [1.07–1.32], **S5 Fig**) and enrichments in protective PTVs in the two other ethnic samples (**S11 Table**). Thus, the lack of overlap in gene identities between these ethnic samples may, in part, be due to the differential sampling of genes whose PT-variants increase T2D risk and genes whose PT-variants decrease risk.

Finally, analogous analyses conducted with the generic-PLN confirmed the increased sensitivity of the T2D-PLN. After constructing gene communities as before, only one community was significantly enriched in the “Mono-Syn” and “GWAS” gene sets (**S12 Table**). There was no enrichment of PTV-associations with this community (**S13 Table**). In fact, the significant community within the generic-PLN (Community 0; 1516 genes) shows an extensive overlap involving 1004 genes with T2D-PLN Community 5 (2390 genes, **S8 Table**) (*p*<10^−16^, hypergeometric test).

### Community 5 genes associated with T2D genetic risk are expressed in the pancreas and liver and are involved in lipid and glucose homeostasis

**Fig 5:**
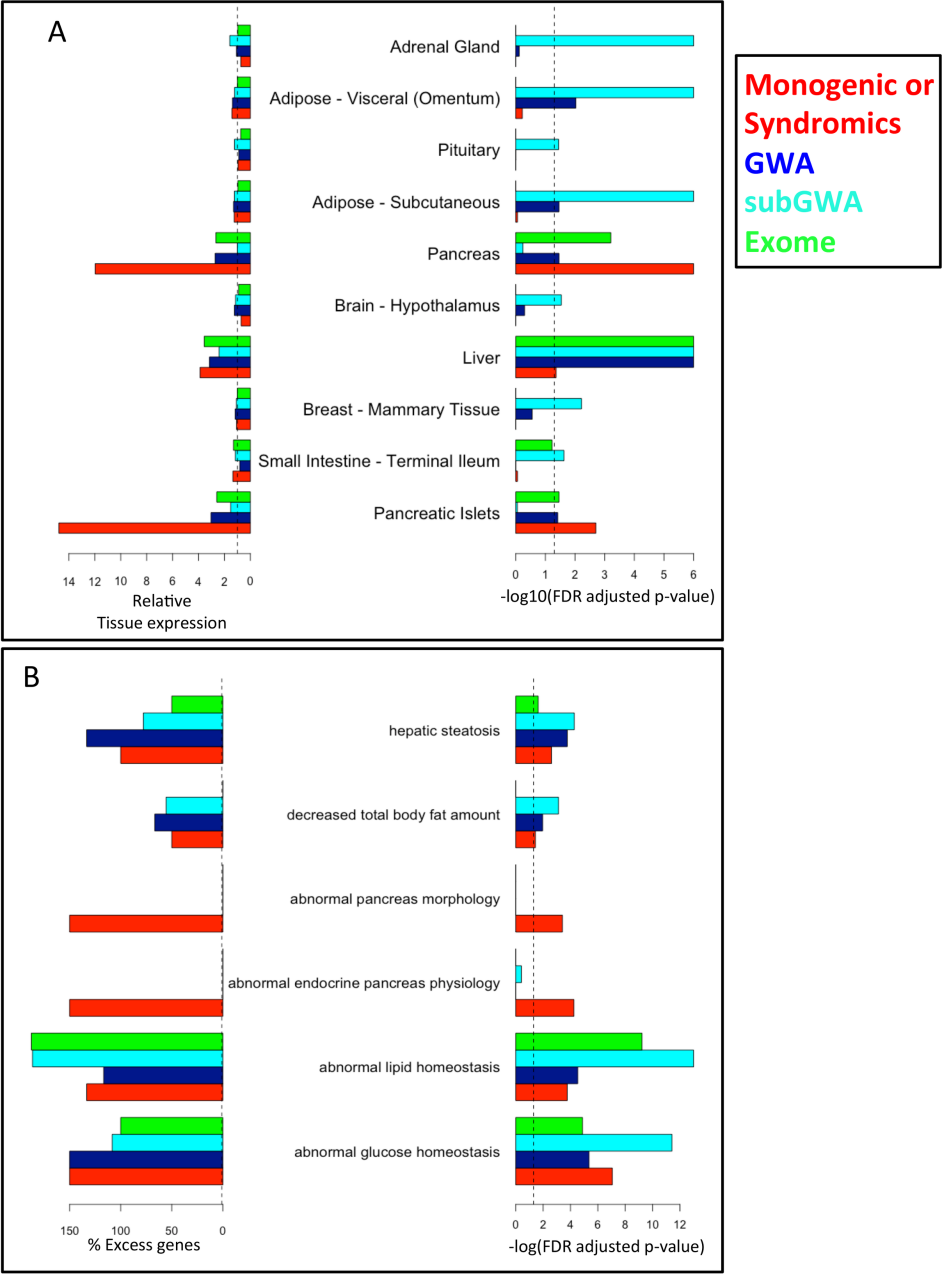
Additional functional annotations of known and candidate T2D-risk genes. **(A)** 10 tissues in which the average expression of genes within at least one of T2D risk set was found to be significantly high. Gene expression with 53 tissues was examined using recently-released data from the GTEx project [26] and transcriptomic profiles in pancreatic islets of 11 individuals [27]. Dotted line represents the significance threshold (FDR < 0.05). Results for all 53 tissues shown in **S6 Fig**. **(B)** Phenotypes enriched following the disruption of the unique mouse orthologues of Monogenic and Syndromic Candidate T2D-risk genes and their corresponding enrichments amongst T2D-risk candidate gene sets. Dotted line represents the significance threshold (FDR < 0.05). Representative phenotypes are shown; All phenotypes shown in **S7 Fig**. (TIF 560 kb).

We considered additional functional genomics resources, which had not been used to generate the T2D-PLN (**S2 Table**) to further elucidate the biological roles of the Community 5 candidate genes, specifically (i) 13 “Mono-Syn” genes, (ii) 71 “GWAS” genes (iii) 500 “subGWAS” genes; and (iv) 168 PTV-associated genes from the four ethnic groups that demonstrated enrichment, excluding genes in latter sets that were members of former sets (**S9 Table**). First, combining Gene Tissue Expression (GTEx) project data[26] combined with human pancreatic islet cell expression dataset[27], we found that all four sets showed high expression in the liver and three of them in pancreatic tissue and islet cells along with other enrichments (**Fig 5A, S6 Fig**). Given these genes’ high expression in islets, we examined and found that “GWAS”, “subGWAS” or PTV-associated gene sets were enriched in genes whose expression was found to be associated with genetic variation in a recently published Expression quantitative trait loci (eQTL) study performed on human islets within Europeans[28] (**S14 Table**; **Methods**). It is notable that the Community 5 PTV-associated genes drawn from the 4 non-European ethnic samples were enriched in eQTL eGenes identified within Europeans.

Finally, we considered the phenotypes observed when the unique 1:1 orthologues of Community 5 associated genes are disrupted in the mouse; These data were used to benchmark other data types in the generation of the T2D-PLN but not included as a data type in the network (**Fig 5B**; **S7 Fig**). The “GWAS”, “subGWAS” and “PTV-associated” gene sets were enriched in a number of mouse phenotypes including: (i) dysfunction in glucose metabolism, e.g. abnormal glucose homeostasis (MP:0002078) (*q* = 4.4x10^-6^, 3.9x10^-12^ 1.3x10^-5^, respectively) and (ii) dysfunction in lipid metabolism e.g. abnormal lipid homeostasis (MP:0002118) (*q* = 3.0x10^-5^, 1,0 x10^-13^, 5.9x10^-10^, respectively) (**S15 Table & S16 Table**).

## Discussion

In this study, we took a PLN, a generalized method for integrating diverse data on gene function weighted in proportion to each data sources’ ability to predict whether genes influence the same mammalian phenotypes, and focused the weighting of information towards only those phenotypes relevant for T2D. The resulting gene network, a T2D-PLN, was able uniquely to identify significant functional clustering both within and between sets of genes associated with T2D through different types of genetic studies (**Fig 3**& **Fig 4**).

In comparison to gene prioritization methods that look for genes that functionally resemble genes previously implicated in the disorder [29] [22, 23], our methodology facilitates a more hypothesis-free approach. Specifically, our approach (i) exploits all genes known to influence the relevant mammalian phenotypes rather than only those previously implicated in the disease, and (ii) identifies candidate disease genes on the basis of an unusual degree of functional convergence amongst genes within a given list, rather than on their functional similarity to previously implicated disease genes [30, 31]. Furthermore, Community 5 within the T2D PLN represents a small fraction of the whole network, and by identifying and then focusing on this sub-network, we focus on processes that are likely most T2D functionally-relevant, representing the most “critical edges” of the network [21].

A phenotype-focused networking approach was able to evidence significant functional convergence amongst genes variants associated with T2D as compared to an unfocussed approach. Genes represented within Community 5 are highlighted through enrichment for signals of T2D association that arise from analyses that are driven by distinct sets of variants, ranging from rare, penetrant alleles (in monogenic and syndromic diabetes), through to common, low effect alleles (from common variant GWAS). Crucially, each of the risk allele discovery strategies had been implemented because of its power to detect a particular category of variants, such that power to detect other variant types is extremely low. Indeed, common variant GWAS tests are not configured to detect associations due to extremely rare alleles; and linkage studies have very poor power for common, low-effect alleles. As a result, each of these enrichment signals can be considered independent evidence contributing to the functional inference, a view reinforced by the observation that each enrichment signal implicates different genes within the convergent network. The limited overlap in the identities of the PTV-associated community 5 gene members contributing to the signals in the four ethnic exome substudies suggests that these signals are the consequence of ethnic-specific variants affecting distinct genes within common pathways and argues for the design of future whole-exome sequencing to be focused on a single population of recent common ancestry. Nonetheless, all four ethnic exome substudies Community 5 members demonstrate convergent clustering with genes from the other T2D risk studies considered here and demonstrate similar expression patterns (**S8 Fig**). Thus, when combining individuals of different ancestries, our results suggest that burden tests performed at the level of the pathway are likely to be more powerful than those focused on specific genes.

While Community 5 contains over two thousand genes, the convergence of these risk genes within this community implicates a smaller, focused subregion in the molecular etiology of T2D. Gene expression analyses showed that the genes contributing to this “subregion” were enriched for expression in relevant tissues; and exploration of mouse phenotype annotations revealed enrichment for highly pertinent functional categories including glucose and lipid homeostasis. Inspection of the genes within these regions allows us to formulate hypotheses regarding processes that may be fundamental to T2D predisposition. For example, the Community 5 subnetwork includes multiple genes encoding mitochondrial enzymes across variant sets (including *GLUD1* (mon-syn), *IDH2* (GWAS), *SIRT3* (exome), *HADHA* (subGWAS) and others; **S8 Fig)** that are highly expressed in beta- cells[27]. The protein products of these genes are located in the mitochondrial matrix and show dense physical interactions with each other. One possible connection to the T2D phenotype of this subnetwork would be via the importance of mitochondrial ATP generation to satisfy the high-energy needs of an active beta-cell. Beyond the pancreas, we note that *PCKS9*, a gene strongly expressed in the liver and involved in lipid homeostasis, is a member of the set of Community 5 PTV genes within South Asian sample (**S5A Fig)**. Population-specific associations with lipid traits have been reported for *PCSK9* [32] while its genetic variants, whose effects are thought to be mimicked by *PCSK9* inhibitors, are associated with lower LDL cholesterol levels and higher T2D risk[33].

Our method to focus the integration of diverse functional genomics data towards only a subset of mammalian phenotypes of specific interest is likely to be a powerful approach to other disorders beyond T2D. Although our data weighting method relies on mammalian genotype/phenotype associations derived from mouse models that are obviously incomplete, ongoing efforts to systematically generate and record mouse model-derived genotype/phenotype associations, along with the increasing number of functional genomics datasets available for integration, promise to significantly increase the power of phenotype-focused functional linkage networks to detect functional convergence.

## Material and methods

### Construction of phenotypic linkage network

The major steps to build a PLN (or T2D-PLN) are as follows:

(1) Design a phenotypic benchmark: In the first step, we construct a mouse phenotypic benchmark from a specified list of mouse phenotype annotations. For the general PLN, we used all available mouse phenotype annotations, while for the T2D-PLN, we considered only T2D mouse relevant phenotype annotations (see Type 2 diabetes relevant phenotypes section). For each pair of genes, we compute the semantic similarity between those genes’ mouse phenotype annotations following the method described by Honti et al. (see Semantic similarity section). Within the benchmark, the number of gene pairs was 15,981,275 and 5,274,124 for general and T2D phenotypic benchmarks, respectively.

(2) Evaluation of each individual dataset on a phenotypic benchmark: to estimate the ability of an individual functional dataset to identify genes more likely to influence the same phenotype, we examine the relation between a score derived from the functional dataset and the semantic phenotypic similarity measure computed in (1). We sort gene pairs according to data-specific scores (supposed proportional to strength of functional information) in descending order. The ordered pairs are divided to bins of 500 gene pairs and the median of the phenotypic semantic similarity scores between gene pairs is calculated and plotted for each bin. The degree of phenotypic similarity expected for random gene pairs is equal to median of all gene pairs. We then use this plot to determine what strength of functional relation (linkage) within each functional dataset is informative to predict phenotypic similarity and rescale the informative part of this last on the phenotypic benchmark.

(3) Re-score of each individual dataset on a phenotypic benchmark: From (2), we define a threshold from which low uninformative linkages derived from a given dataset are identified and informative bins are defined as those above the overall median of the semantic phenotypic similarity measure. Above this threshold, we fit polynomial regression (PR) curves in order to re-score the links so that any data-specific scores characterising the gene pairs are replaced with the semantic similarity of phenotypes that they correspond to according to a linear regression function. To determine the degree of the PR, we compared a PR of order n+1 with a PR of order n with an Analysis of Variance (ANOVA). We considered that the polynomial regression of order n+1 does fit better than polynomial regression of order n, when the Pr (>F) was greater than 1%.

(4) Integration of re-scaled functional dataset into single phenotypic linkage genes network: In the case, where different genomic datasets suggest a functional link between the same gene pairs, the functional measures of different datasets are summed, by penalizing the less reliable data according to a formula proposed by Lee et al. [34]:

$$WS=L_{o}+\sum_{i=1}^{n}\frac{L_{i}}{D\times i}$$,

where L represents a re-scored functional measure from a single data set, L_0_ being the largest functional measure among all the functional datasets between the given two genes, i is the index of the remaining links ordered by their weights for the gene pair and D is a free parameter. To define the D parameter that is the best linear predictor a phenotypic semantic similarity measure, we sorted gene pairs according to WS scores calculated with specific D parameter in descending order. The ordered pairs are divided to bins of 500 gene pairs and the median of the phenotypic semantic similarity scores was calculated and plotted for each bin. We fitted a simple linear regression function to model the relation between phenotypic similarity measure and each WS associated with a specific D parameter (D value from 1 to 20) and determined which *WS*_*D*_ was associated with low P(Pf>F) in an ANOVA test for regression. We determined D = 6 and D = 7 for the PLN and T2D-PLN respectively.

### Type 2 diabetes relevant phenotypes

Nicola Beer (N.B.) defined a list of mouse and human phenotypes relevant to T2D from the phenotypes listed within the Mouse Phenotype Ontology (MPO)[35] (http://www.informatics.jax.org/). The MPO phenotypes are organized into 33 over-arching categories, and formalized in an ontological hierarchy, where more specific child terms logically inherit the annotations of those terms more general parent terms. The 38 mouse phenotypes deemed relevant to T2D (**S1 Table**) fall into three over-arching categories, specifically endocrine/exocrine gland phenotype (MP:0005379), homeostasis/metabolism phenotype (MP:0005376) and adipose tissue phenotype (MP:0005375). In order to provide a sufficient number of gene pairs with which to reliably benchmark the ability of different genomics resources to identify phenotypic similarities, we considered all 3588 genes associated with these 3 over-arching phenotypes when evaluating datasets for inclusion in the T2D-PLN.

### Datasets used to build the general PLN and T2D-PLN

Mouse phenotype annotation to build a phenotypic benchmark: we used the Mammalian Phenotype Vocabulary in OBO v1.2 and Mouse/Human Orthology with Phenotype Annotations that we downloaded here: http://www.informatics.jax.org/downloads/reports/index.html

Datasets integrated in the final T2D-PLN and PLN: We re-used four of eight co-expression dataset namely: GNF2[36], GSE3594 [37], MTAB-62[38], Shyamsundar et al. (2005) [39] and one novel co-expression dataset Iacobuzio (2003) coming from [40]. We calculated the Pearson's correlation coefficient of their expression profiles, to estimate the co-expression of genes. We then used exactly the same datasets as Honti et al. [13] to build both the general PLN and T2D-PLN namely: Gene annotations from Gene Ontology using the annotations of human and mouse genes in the Biological Process (BP), Molecular Function (MF) and Cellular Component (CC) categories [41], the same co-citation scores [42] (literature), protein-protein interaction dataset (section), Kegg pathway annotations of mouse genes [43], pathway annotations of human genes from Reactome [44], protein domain annotations from InterPro [45]. For all gene annotations, GO, KEGG, Reactome, InterPro, we employed the same approach than Honti *et al*. to compute semantic similarity measures [13].

The general PLN and T2D-PLN included 16044620 and 7811440 of weighted links (**S2 Table**) respectively. T2D-PLN was built by evaluating the same 15 individual datasets (**Fig 1**) as the PLN (**S2 Table**), but on the T2D phenotypic benchmark. Thereafter, we considered only the 1,000,000 highest weighted links of different PLN (T2D and unspecific PLN networks): we found that 1,000,000 highest weighted links were informative to predict human phenotype similarity between two genes (**S9 Fig**).

### Testing for functional clustering of a gene-set

To evaluate whether a given set of genes demonstrated unusually similar functionality, we examined the extent to which those genes clustered within the specified phenotypic-linkage network (PLN or T2D-PLN). For this, we compared the sum of weighted links observed between these genes as compared to an equal number of random genes and computed an empirical p-value. As the coding-sequence (CDS) length and the network connectivity (degree) can bias functional network associations[24, 46], the randomized genes are matched in CDS length and degree to the original genes.

### GWAS and subGWAS interval definition

The T2D GWAS intervals were defined, by identifying the most distant pair of SNPs with r^2^ > 0.5 in 1000 Genomes Phase I data[47], using the appropriate continental subset of 1000 Genomes samples, by extending then each region of interest by moving out 0.02 cM, to encompass nearby recombination hotspots, and by adding an additional 300kb upstream and downstream. We merged overlapping intervals and excluded 10 regions encompassing one of the genes associated with monogenic and syndromic forms of diabetes (**S3 Table)**, yielding 72 unique associated regions (**S4 Table**), and identified 579 genes completely or partially included within associated regions.

The T2D subGWAS intervals were defined by considering SNPs attaining an association p-value<10^−3^ in a recently-completed GWAS meta-analysis of mostly European individuals[4] (http://diagram-consortium.org) by identifying the most distant pair of SNPs with r^2^ > 0.5 computed by considering the European haplotype in the 1000 Genome Project and then adding an additional 250 kb on either side of the interval to include genes that may be regulated by GWAS-associated regulatory variants. We identified 613 subGWAS intervals encompassing 4150 protein coding genes, by discarding subGWAS interval overlapping the 72 T2D risk GWAS intervals and any intervals or encompassing Mono-Syn genes.

### Network prediction of T2D genes from 32 monogenic and syndromic diabetes genes

We considered 32 “Mono-Syn” genes (**S3 Table**). We diffused this information through an integrative functional network to identify new T2D genes employing an iterative ranking algorithm[48], where the score of each gene to be associated with T2D pathway depends on its neighbors such:

$$f^{t+1}=\alpha f^{0}+(1-\alpha )Uf^{t}$$,

where f^t^, α, U correspond to score of each gene at time t (100 iterations performed), “back probability” (set here 0.5) expresses the probability of jumping to the initial node at each iteration and the matrix of normalized weighted links respectively. The network diffusion approach enables us to take into account genes that are indirectly connected with seed genes. To evaluate the significance of scores associated with each gene, we compared these score with the distribution of scores obtained by sampling 32 random seed genes and calculated an empirical p-value named p-value_mono_.

### Detection of functional relationships between monogenic and syndromic diabetes genes and gene lying GWAS or subGWAS region

We compared the number of genes lying GWAS/subGWAS intervals (see definition) and linked within our PLN/T2D-PLN with Mono-Syn genes (we used an p-value_mono_ <0.05), with the same metric in shifted GWAS (subGWAS) intervals and computed the empirical p-value associated, which was associated with significance degree of linkage between Mono-Syn genes and genes lying within subGWAS and GWAS regions (**Fig 2**). In order to take into account genome clustering [49] and conserved the same number of genes than in the observed GWAS (subGWAS) intervals, the GWAS and subGWAS intervals have been shifted 100,000 times.

### Identification of network communities

To identify the different gene communities, we employed a greedy algorithm suited to a large network, specifically the Louvain algorithm[50] [51], which is based on the Potts method. This approach involves two steps: (1) the identification of clustered gene modules (2) and the iterative aggregation of additional genes into these modules. The iterations are repeated until the modularity does not increase. We used the Louvain method implemented in Gephi to identify the genes network community (version 0.8.2 beta)[52].

The resolution parameter, set to 1 in this study determines the degree of modularity: the number of communities decreases when the resolution parameter increases (**S10 Fig**). We found that a resolution parameter ~ 1 maximizes the network modularity (**S11 Fig**). Setting this resolution parameter, we identified 374 and 228 communities containing respectively between 2 and 2390 genes and between 2 and 2287 within the generic PLN and T2D-PLN.

### Identification of specific functional communities of genes significantly enriched within Mon-Syn and PTV gene sets

We considered only those 18 communities that were formed from at least 20 genes and thus able to provide reasonable power for subsequent analyses. To evaluate whether a specific community was enriched in a given set of genes (“Mono-Syn” (**S5 Table & S12 Table**) and PTVs genes (**S6 Table**)), we compared the number of genes belonging to this community as compared to an equal number of random genes and calculated an empirical p-value. The randomly-selected genes are matched in CDS length to the original genes. We adjusted the nominal p-value using a Bonferroni correction for the “Mono-Syn” set and an FDR correction for PTVs gene sets.

### Identification of specific functional communities of genes significantly enriched within T2D GWAS interval genes

We considered only those 18 communities that were formed from at least 20 genes and thus able to provide reasonable power for subsequent analyses. Taking each of the 18 communities in turn and considering 72 GWAS intervals or 613 subGWAS intervals (**S5 Table** & **S12 Table**), we examined how many of these intervals harboured at least one gene belonging to that community as compared to randomly shifted intervals of equal gene number (10,000 random sets) and calculated an empirical p-value, which was then adjusted by Bonferroni correction.

### Risk evaluation in the different ethnic samples of different genes set impacted by T2D PTV

For 40, 51, 42 and 45 genes impacted by T2D-risk PT-variants in the African-American, East-Asian,Hispanic and South-Asian ethnic samples and belonging to the Community 5, we computed a burden p-value in each ethnic sample, by comparing the sum of -log10 SNP-set (Sequence) Kernel Association Test–O (SKAT-O) of their SKAT-O test risk association p-value computed in the original study [8] with those of random genes matching T2D PTVS genes for their CDS length and calculated an empirical p-value that we adjusted by using an Bonferroni correction (**S10 Table, S12 Fig**).

### Evaluation of the effects of PT-variants on T2D risk

To evaluate the effect of PT-variants of a gene set on T2D risk in a given ethnic group of individuals, we performed a burden test in which we compared the relative number of PT-variant mutations between case and control groups as follows:

$$d_{obs}=\frac{x_{cases}}{n_{cases}}-\frac{x_{controls}}{n_{controls}},$$

where x and represents the number of PT-variants in a given gene for the stated group and n is the total number of individuals in each group. We computed an empirical p-value associated with d_obs_ by permuting cases and controls status between individuals (**S11 Table).**

We also evaluated the effect of PT-variants within a given gene set on T2D risk using the following logistic regression model:

$$Logit\{Y=1|X\}=\beta_{0}+\beta_{1}X$$

where Y is the phenotypic vector (disease status) and X is a vector describing for each individual the number of PT-variants in a given gene set. A T2D risk measure of PTVS affecting a set of gene is computed with following Odds Ratio (OR) estimation: *OR* = exp(*β*_1_) (**S11 Table).**

### Evaluation of tissue specificity of T2D associated genes

53 tissues from Genotype-Tissue Expression (GTEx) (http://www.gtexportal.org/)[26] and additionally pancreatic islet expression of seven samples were used [27]. For each protein coding gene, we computed the mean of Reads Per Kilobase of transcript per Million mapped reads. (RPKM) values across all samples for each tissue in turn. We ranked each gene according to their RPKM value within each tissue. We scaled rank values to between 0 and 1 for each tissue. We performed an Euclidean normalization of scaled rank values of each gene to get tissue specificity vectors reflecting whether one gene is expressed in a given tissue but not in others. To evaluate the tissue specificity of a gene set for a given tissue or cell type, we compared the sum of normalized tissue expression with those of random gene sets matching the query genes for the gene length to derive an empirical p-value, which was adjusted by using False discovery rate (FDR) correction.

### Network genes representations

Network representations were made with Gephi (version 0.8.2 beta)[52].

### Data availability

Our general and T2D phenotypic-linkage network used in this study are available here: http://wwwfgu.anat.ox.ac.uk/downloads/compbio_projects/CW003_SANDOR_T2D.

## Supporting information

S1 Fig T2D-PLN statistics. The left and right represent the distribution of number of links and sum of weighted links by gene respectively. The red vertical line corresponds to mean of each distribution.
(TIF)

S2 Fig Genes within 72 T2D risk known GWAS intervals that are functionally-linked by PLNs to other T2D-risk variants considered in this study. The outer circle represents 72 GWAS intervals where the gene closest to the lead SNP in each interval is denoted by a red line, while other genes revealed by a T2D-PLN or a PLN are denoted by black bars. The inner circles represent the genes functionally associated with 29 mono genes by a PLN (gray circle) and a T2D-PLN (blue circle), and those genes that belong to T2D-PLN Communitiy 5 (red circle).
(TIF)

S3 Fig Venn diagram showing the overlap between Community 5 genes affected by T2D-risk protein-truncating variants (300 most risk-associated genes based on SKAT-O test p-value) in different ethnic samples. (TIF)

S4 Fig Conserved juxtapositions of sets of known and candidate T2D-risk genes within a sets-combined network, and their functional associations within the T2D-PLN Community 5. Each named dot represents a known (panel A) or candidate (panels B-G) T2D-risk genes. Panel A: 13 monogenic and syndromic (Mono). Panel B: 71 genes residing within 72 T2D-risk GWAS intervals. Panels C-G: 40, 42, 51, 45 and 168 genes impacted by T2D-risk PT-variants in the African-American, Hispanic, East-Asian, South-Asian samples and then all samples except the [non-significant] European sample respectively. The colour of the link connecting two genes indicates the strongest information source supporting the functional association.
(TIF)

S5 Fig PT-variant case-control frequencies across 4 ethnic samples for the South-Asian Community 5 PT-variant genes that present either risk or protective effects in the South-Asian sample. (TIF)

S6 Fig Tissue expression enrichment analyses for different class of T2D-risk genes. The average expression level of genes for each known or candidate T2D-risk gene set relative to other genes in pancreatic islet[27] and in 53 tissues reported by the GTEx project [26].
(TIF)

S7 Fig Mouse phenotype enrichment analyses. Phenotypes enriched following the disruption of the unique mouse orthologues of Monogenic and Syndromic Candidate T2D-risk genes and their corresponding enrichments amongst T2D-risk candidate gene sets. Dotted line represents the significance threshold (FDR < 0.05).
(TIF)

S8 Fig Tissue expression enrichment analyses for Community 5 genes set impacted by T2D-risk PT-variants in different ethnic-samples. The average expression level of genes for each known or candidate T2D-risk gene set relative to other genes in pancreatic islet [27] and in 53 tissues reported by the GTEx project [26].
(TIF)

S9 Fig Annotations of regulatory interactions by Ingenuity Pathway Analysis. (IPA, QIAGEN Redwood City, **www.qiagen.com/ingenuity**) of 16 Community 5 genes contributing to a PPI network within mitochondria and that are highly expressed in beta cells. IPA annotations were performed considering the following genes identified by T2D-PLN network: *HADH*, *GLUD1* ("Mono-Syn"; red), IDH2 ("GWAS"; dark blue), *HADHB*, *HIBCH*, *GSR*, *ME3*, *DLAT*, *HSD3B2*, *HSD3B1*, *HADHA*, *AASS*, *ABCF2* ("subGWAS"; cyan), *CAT*, *ALDH1B1*, *SIRT3* ("Exome”; green). IPA ascribes lipid metabolism as the function. The relationship of annotated edges are: A activation, E expression, I Inhibition, LO Localization, M Biochemical Modification, MB Group/complex Membership, PP Protein-Protein Binding, RB Regulation of Binding, T Transcription, TR Translocation.
(TIF)

S10 Fig Ability of the PLN and T2D-PLN networks to predict the pairwise similarity of Human Phenotype Ontology annotations assigned to human genes. The median of semantic similarity score between gene pairs was determined from their human phenotype annotations database (http://www.human-phenotype-ontology.org/) [53]. Gene pairs were sorted according to the weight of their link either within the un-specific PLN (black curve) or the T2D-PLN (blue curve). One point represents a bin of 500 gene pairs.
(TIF)

S11 Fig The number of gene communities formed according to the resolution parameter employed in the Louvain algorithm. The gray and blue curves represent the number of communities formed when clustering the un-specific PLN and T2D-PLN network, respectively.
(TIF)

S12 Fig Network modularity according to the resolution. (TIF)

S13 Fig Community 5 PT-variant genes SKAT-O loadings across all 4 ethnic samples. For 40, 42, 51, 45 Community 5 genes impacted by T2D-risk PT-variants in the African-American, Hispanic, East-Asian, South-Asian samples, respectively (rows), we report the–log10 of p-value associations from the SKAT-O tests for these genes within each of four ethnic samples (four points per row, following the Y-axis ethnic sample colourings.).
(TIF)

S1 Table Mouse phenotypes annotations relevant for T2D defined by NLB. The mouse T2D phenotypes were selected from phenotypes annotations from the Mouse Genome Database (http://www.informatics.jax.org/).
(XLSX)

S2 Table Coverage of gene pairs within general PLN and T2D-PLN for each different individual dataset. (XLSX)

S3 Table List of monogenic and syndromic diabetes genes. (XLSX)

S4 Table 72 T2D GWAS intervals that do not include monogenic and syndromic diabetes genes. (XLSX)

S5 Table Evaluation of enrichment for T2D-PLN genes communities in “Mono-Syn”, “GWAS” and “subGWAS” genes. (XLSX)

S6 Table Enrichment test of gene communities in T2D-PLN for genes including T2D risk exome variants. (XLSX)

S7 Table Effect of varying the number of considered genes impacted by PT exome-variants selected for the enrichment test of gene communities in the T2D PLN. (XLSX)

S8 Table Ensemble IDs of all genes in T2D-PLN community 5. (XLSX)

S9 Table Sets of known and candidate T2D-risk genes within the T2D-PLN Community 5. (XLSX)

S10 Table Ethnic-specific PTV variants affecting distinct genes within common pathways. For 40, 51, 42 and 45 genes impacted by T2D-risk PT-variants in the African-American, East-Asian, Hispanic and South-Asian ethnic sample and belonging to the Community 5, for each ethnic sample we computed a burden p-value (value in the cell), by comparing the sum of -log10 S of their SKAT-0 test risk association p-value with those of random genes matching T2D PTVS genes for their CDS length and calculated an empiric p-value, which was then adjusted by using an Bonferroni correction. The orange and gray cells represent a significant and un-significant enrichment in T2D-risk PT variants respectively.
(XLSX)

S11 Table Evaluation of the effect of PT-variant impacting genes of the Community 5 on T2D risk. To evaluate the effect of PT-variants of a genes set on T2D risk in a given ethnic group of individuals, we performed a burden test in which we compared the relative number of PT-variant mutations between case and control (left, d_obs_ (see Methods)) and by using a logistic regression odds ratio (right). We examined 40, 51, 42 and 45 genes impacted by T2D-risk PT-variants in the African-American, East-Asian,Hispanic and South-Asian ethnic sample and belonging to the Community 5 (top) and also 170 genes impacted by T2D-risk PT-variants in the four previous ethnic sample and belonging to the Community 5 (bottom).
(XLSX)

S12 Table Evaluation of enrichment for the generic PLN genes communities in “Mono-Syn” and “GWAS”. (XLSX)

S13 Table Table: Enrichment test of gene communities in the generic PLN for genes impacted by protein truncating T2D risk exome variants. (XLSX)

S14 Table Enrichement analyses for different T2D-risk candidate gene sets in genes whose expression in human islets was found to be associated with genetic variation. (XLSX)

S15 Table Mouse phenotype enrichment for GWAS genes in the Community 5. (XLSX)

S16 Table Table: Mouse phenotype enrichment for genes impacted by PT-variants in the Community 5. (XLSX)
