# Supplementary figures and images for: Diverse type 2 diabetes genetic risk factors functionally converge in a phenotype-focused gene network
Source: PLoS Comput Biol. 2017 Oct 23;13(10):e1005816. doi: 10.1371/journal.pcbi.1005816 (PMC5667928; doi:10.1371/journal.pcbi.1005816)

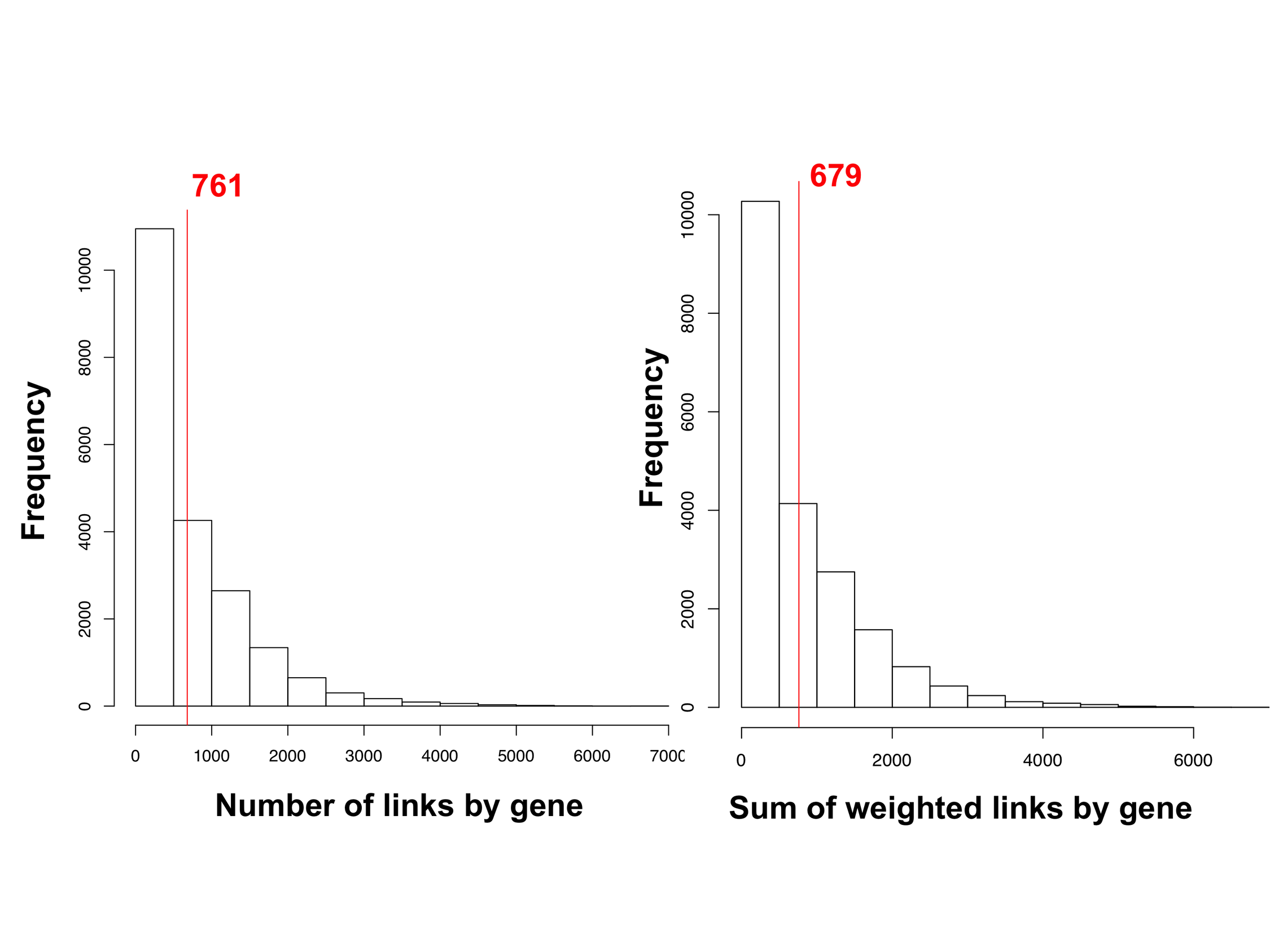

Supplement: S1 Fig — The left and right represent the distribution of number of links and sum of weighted links by gene respectively. The red vertical line corresponds to mean of each distribution. (TIF) [file pcbi.1005816.s001.tif]

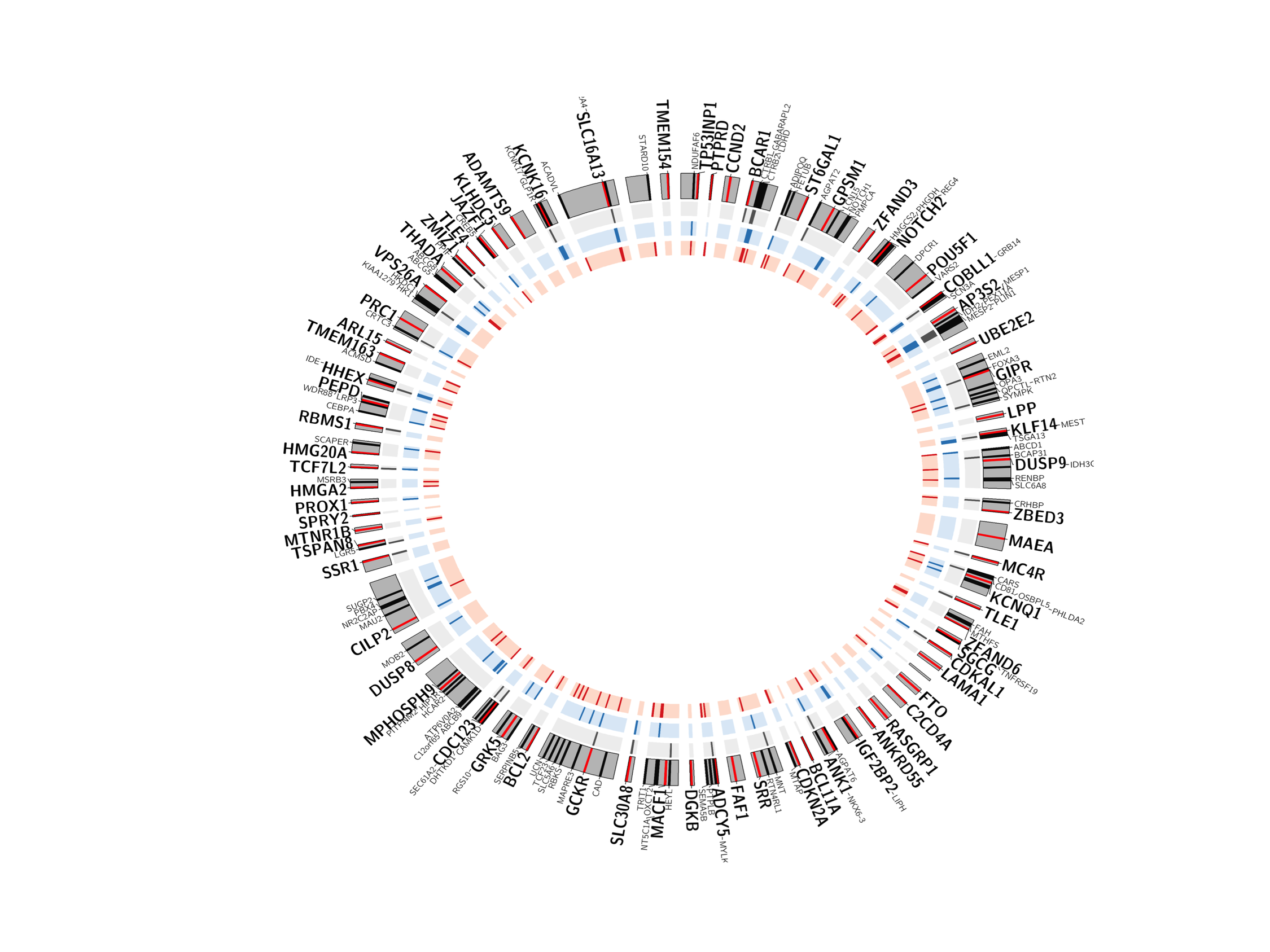

Supplement: S2 Fig — The outer circle represents 72 GWAS intervals where the gene closest to the lead SNP in each interval is denoted by a red line, while other genes revealed by a T2D-PLN or a PLN are denoted by black bars. The inner circles represent the genes functionally associated with 29 mono genes by a PLN (gray circle) and a T2D-PLN (blue circle), and those genes that belong to T2D-PLN Communitiy 5 (red circle). (TIF) [file pcbi.1005816.s002.tif]

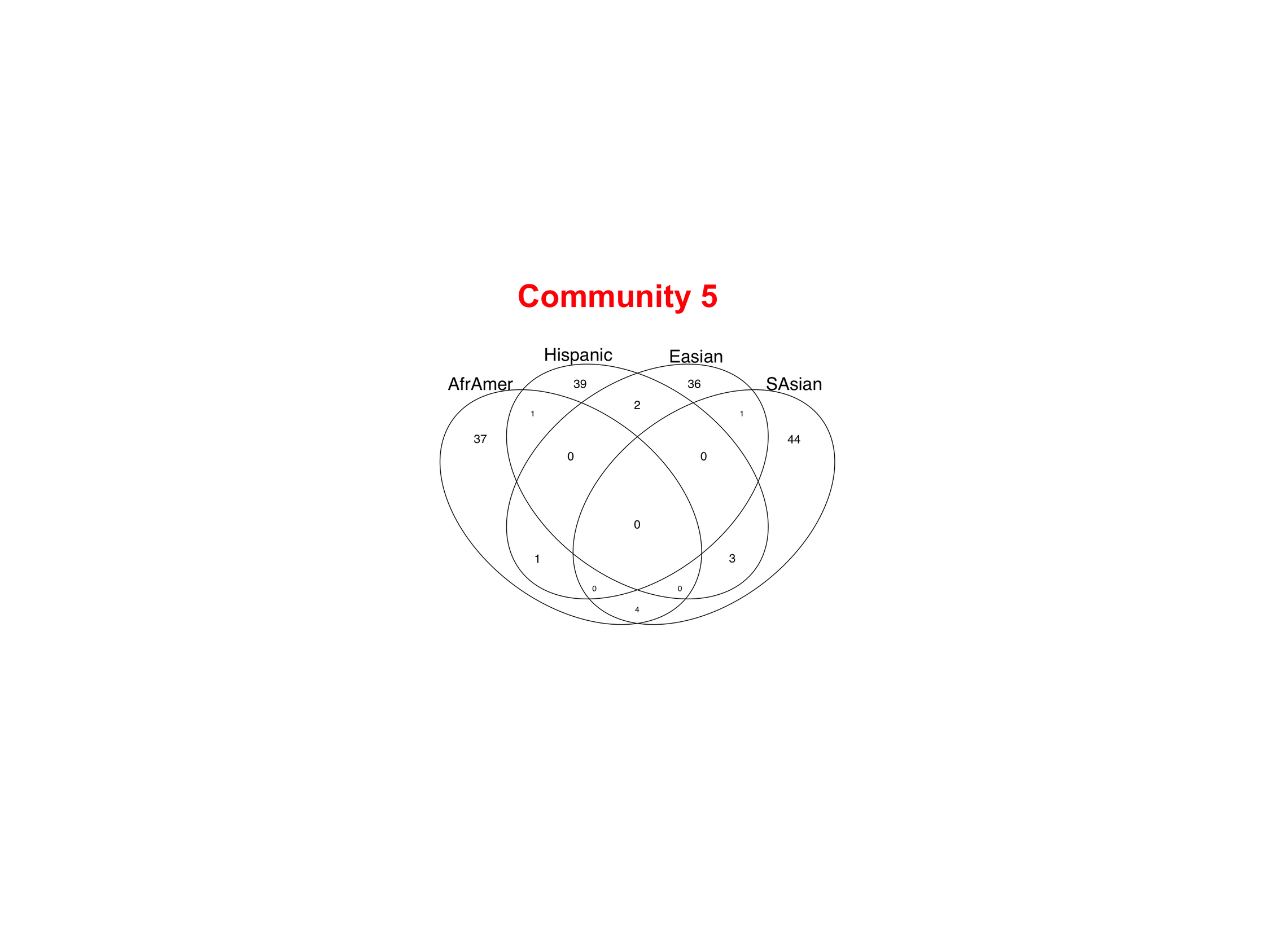

Supplement: S3 Fig — (TIF) [file pcbi.1005816.s003.tif]

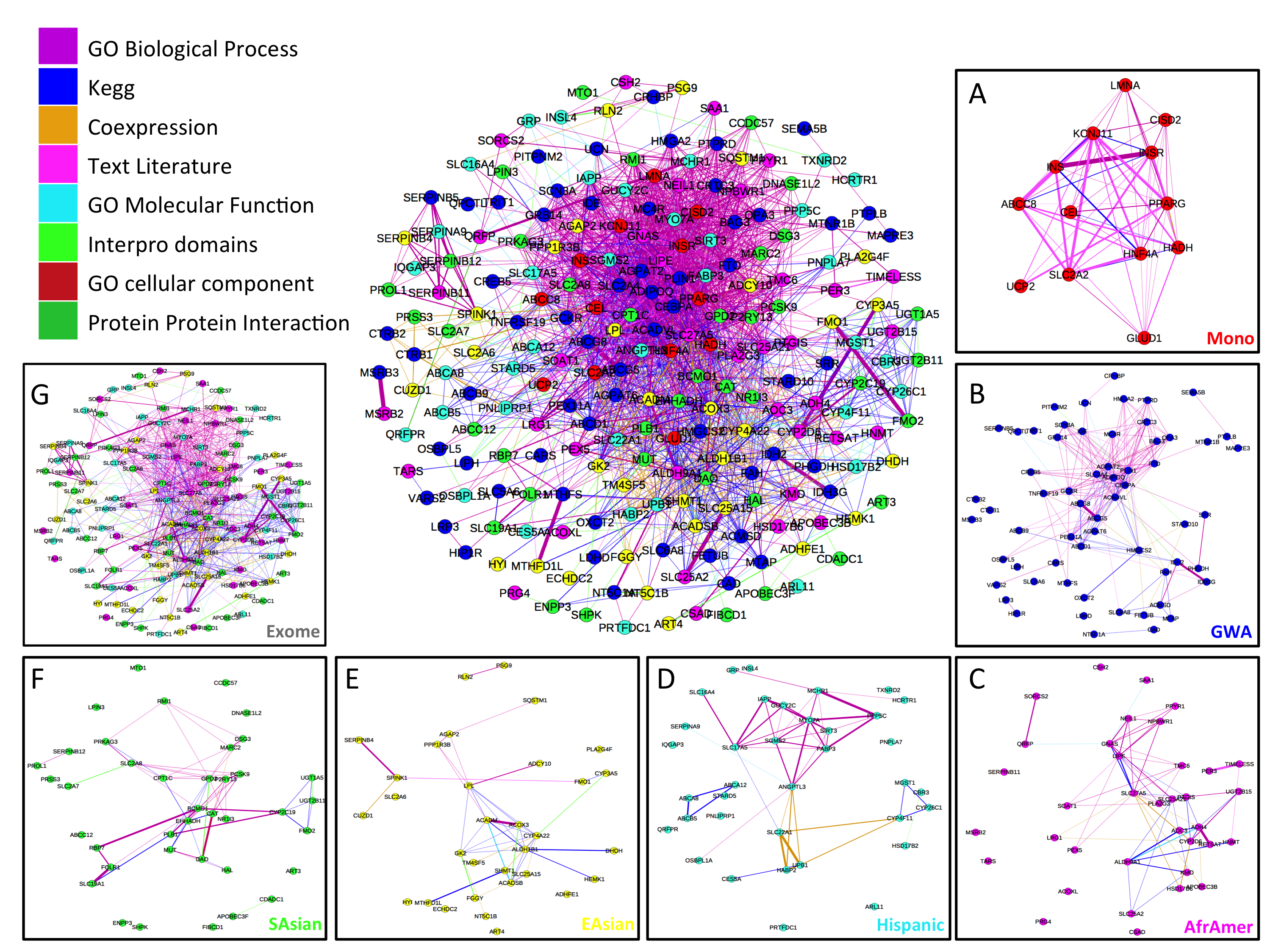

Supplement: S4 Fig — Each named dot represents a known (panel A) or candidate (panels B-G) T2D-risk genes. Panel A: 13 monogenic and syndromic (Mono). Panel B: 71 genes residing within 72 T2D-risk GWAS intervals. Panels C-G: 40, 42, 51, 45 and 168 genes impacted by T2D-risk PT-variants in the African-American, Hispanic, East-Asian, South-Asian samples and then all samples except the [non-significant] European sample respectively. The colour of the link connecting two genes indicates the strongest information source supporting the functional association. (TIF) [file pcbi.1005816.s004.tif]

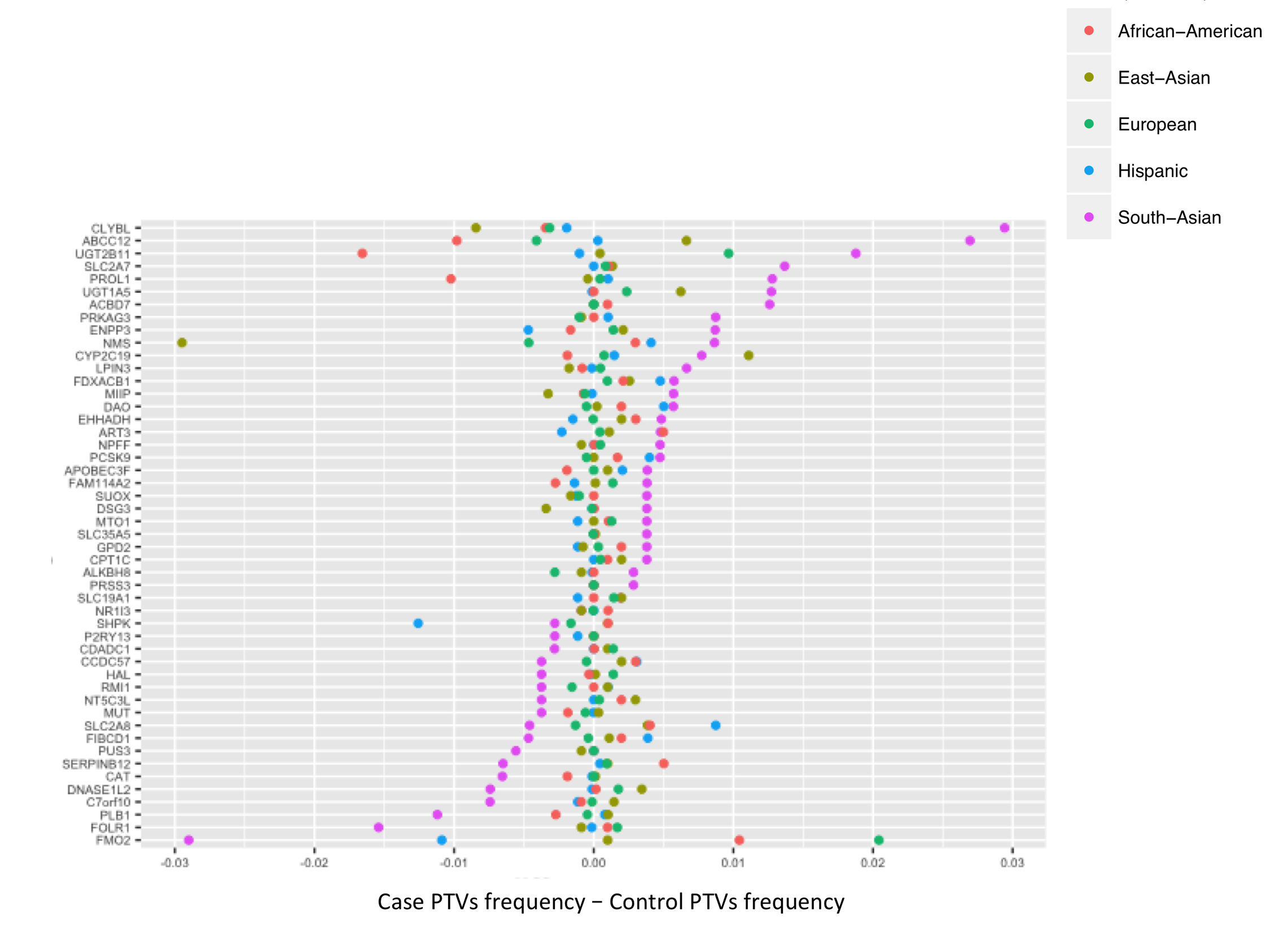

Supplement: S5 Fig — (TIF) [file pcbi.1005816.s005.tif]

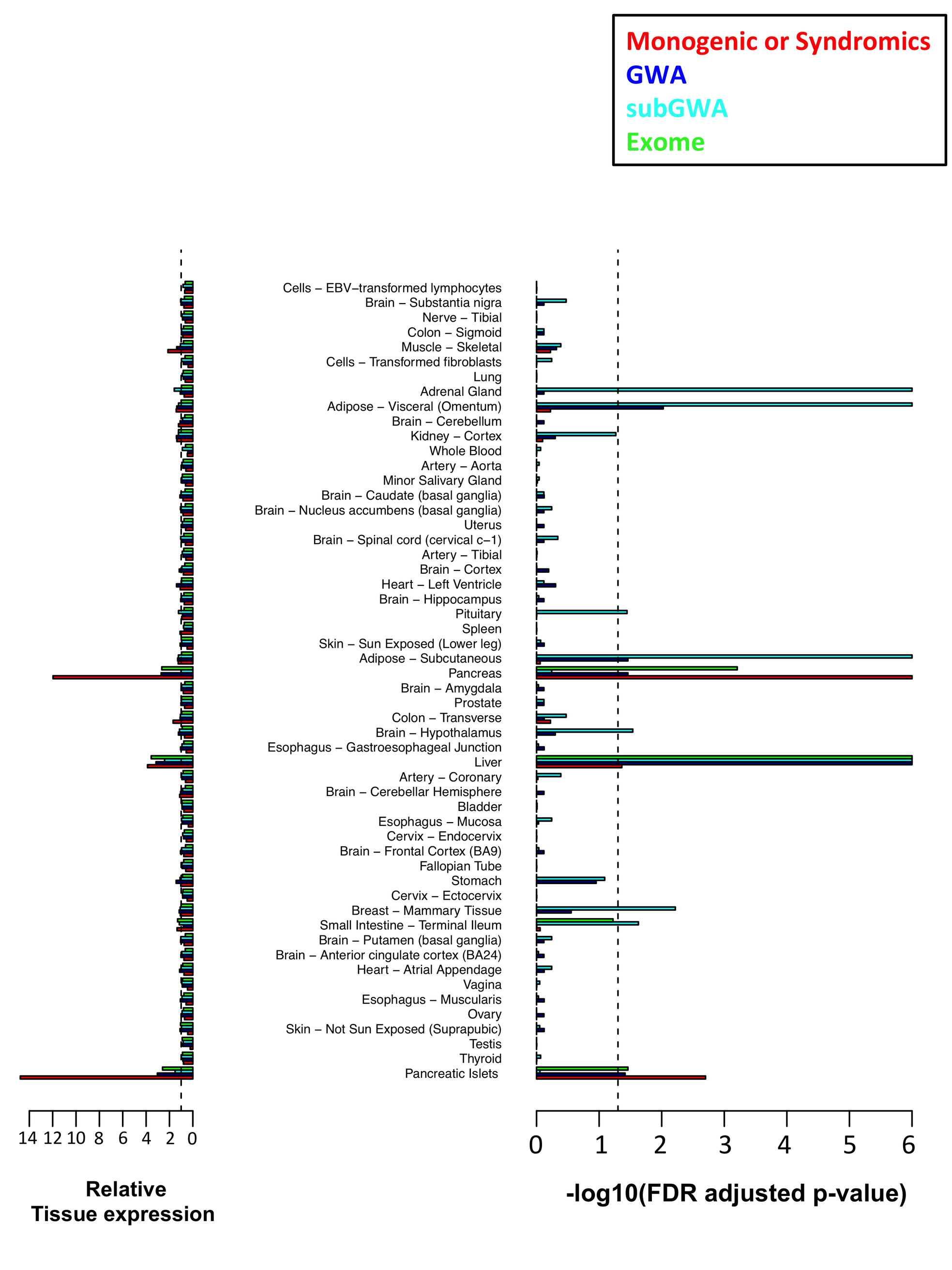

Supplement: S6 Fig — The average expression level of genes for each known or candidate T2D-risk gene set relative to other genes in pancreatic islet[27] and in 53 tissues reported by the GTEx project [26]. (TIF) [file pcbi.1005816.s006.tif]

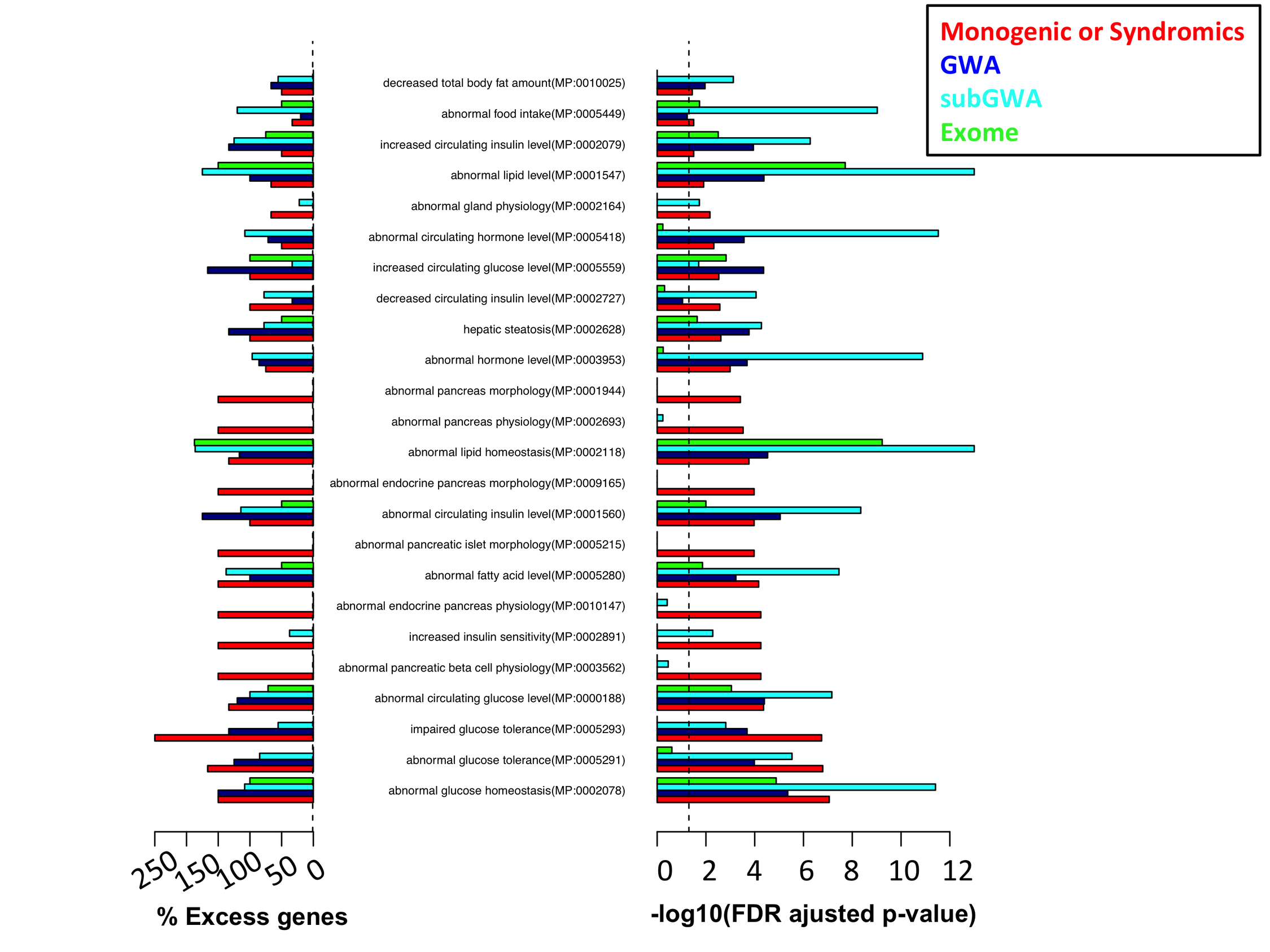

Supplement: S7 Fig — Phenotypes enriched following the disruption of the unique mouse orthologues of Monogenic and Syndromic Candidate T2D-risk genes and their corresponding enrichments amongst T2D-risk candidate gene sets. Dotted line represents the significance threshold (FDR < 0.05). (TIF) [file pcbi.1005816.s007.tif]

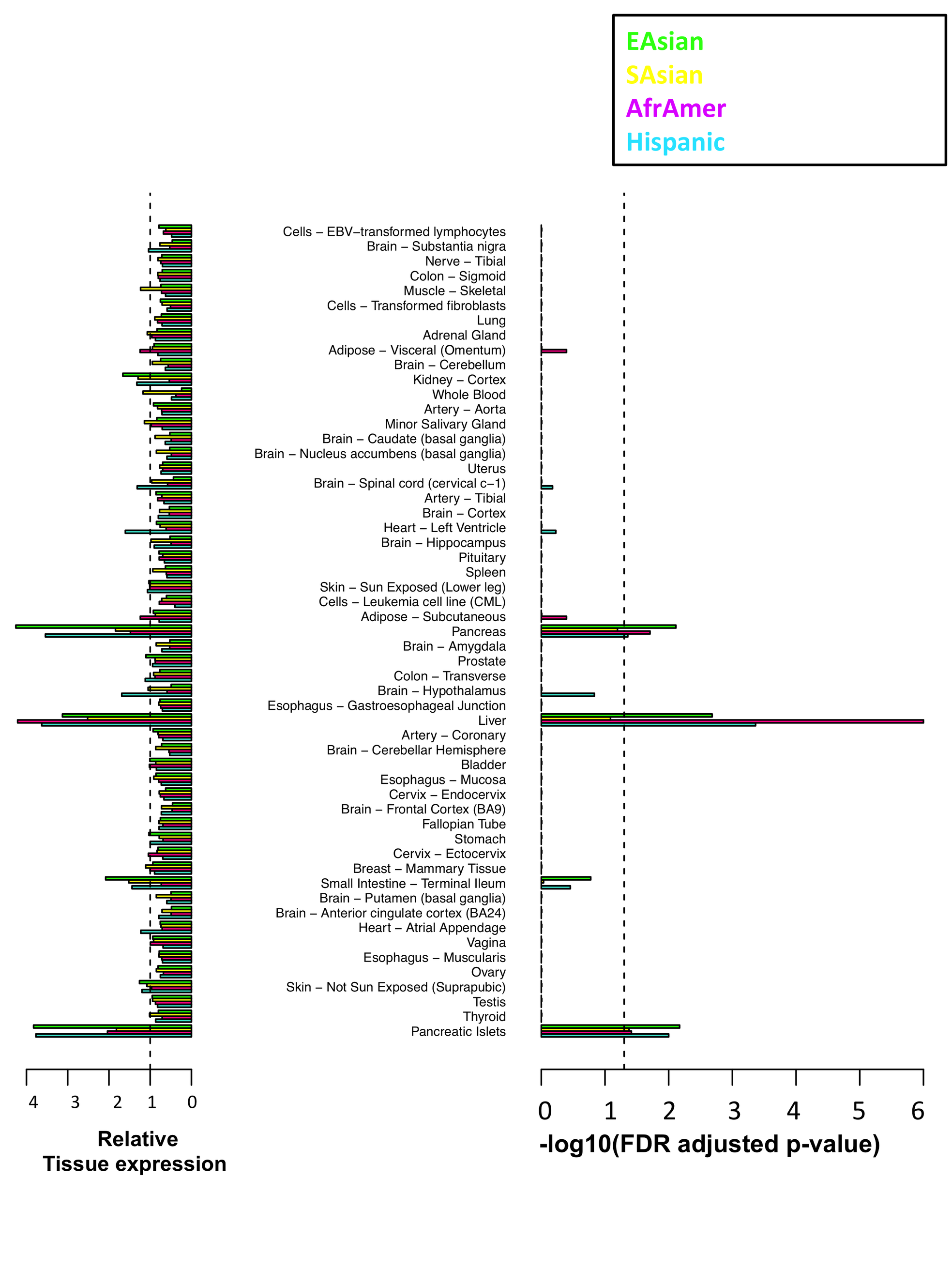

Supplement: S8 Fig — The average expression level of genes for each known or candidate T2D-risk gene set relative to other genes in pancreatic islet [27] and in 53 tissues reported by the GTEx project [26]. (TIF) [file pcbi.1005816.s008.tif]

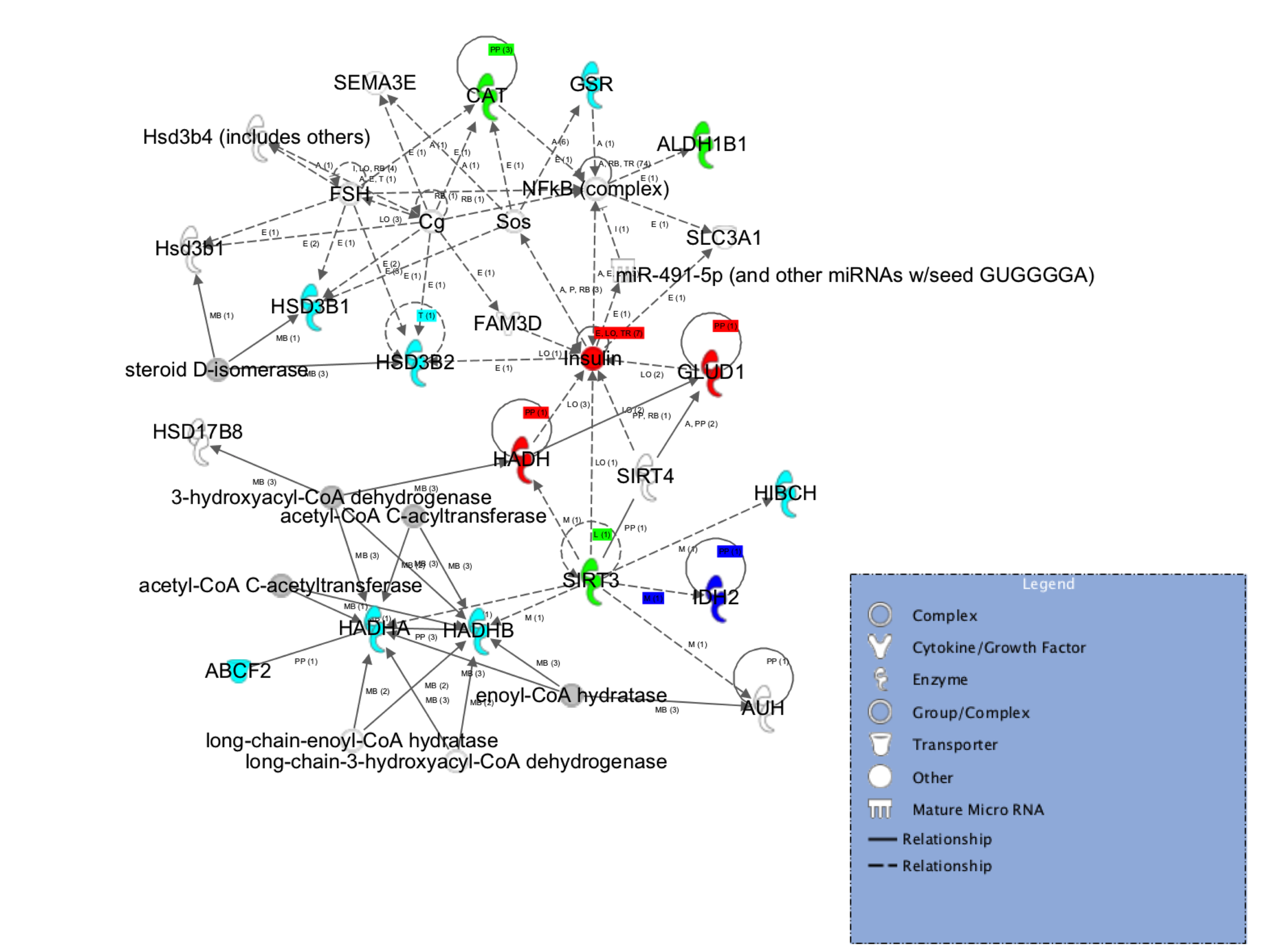

Supplement: S9 Fig — (IPA, QIAGEN Redwood City, www.qiagen.com/ingenuity) of 16 Community 5 genes contributing to a PPI network within mitochondria and that are highly expressed in beta cells. IPA annotations were performed considering the following genes identified by T2D-PLN network: HADH, GLUD1 ("Mono-Syn"; red), IDH2 ("GWAS"; dark blue), HADHB, HIBCH, GSR, ME3, DLAT, HSD3B2, HSD3B1, HADHA, AASS, ABCF2 ("subGWAS"; cyan), CAT, ALDH1B1, SIRT3 ("Exome”; green). IPA ascribes lipid metabolism as the function. The relationship of annotated edges are: A activation, E expression, I Inhibition, LO Localization, M Biochemical Modification, MB Group/complex Membership, PP Protein-Protein Binding, RB Regulation of Binding, T Transcription, TR Translocation. (TIF) [file pcbi.1005816.s009.tif]

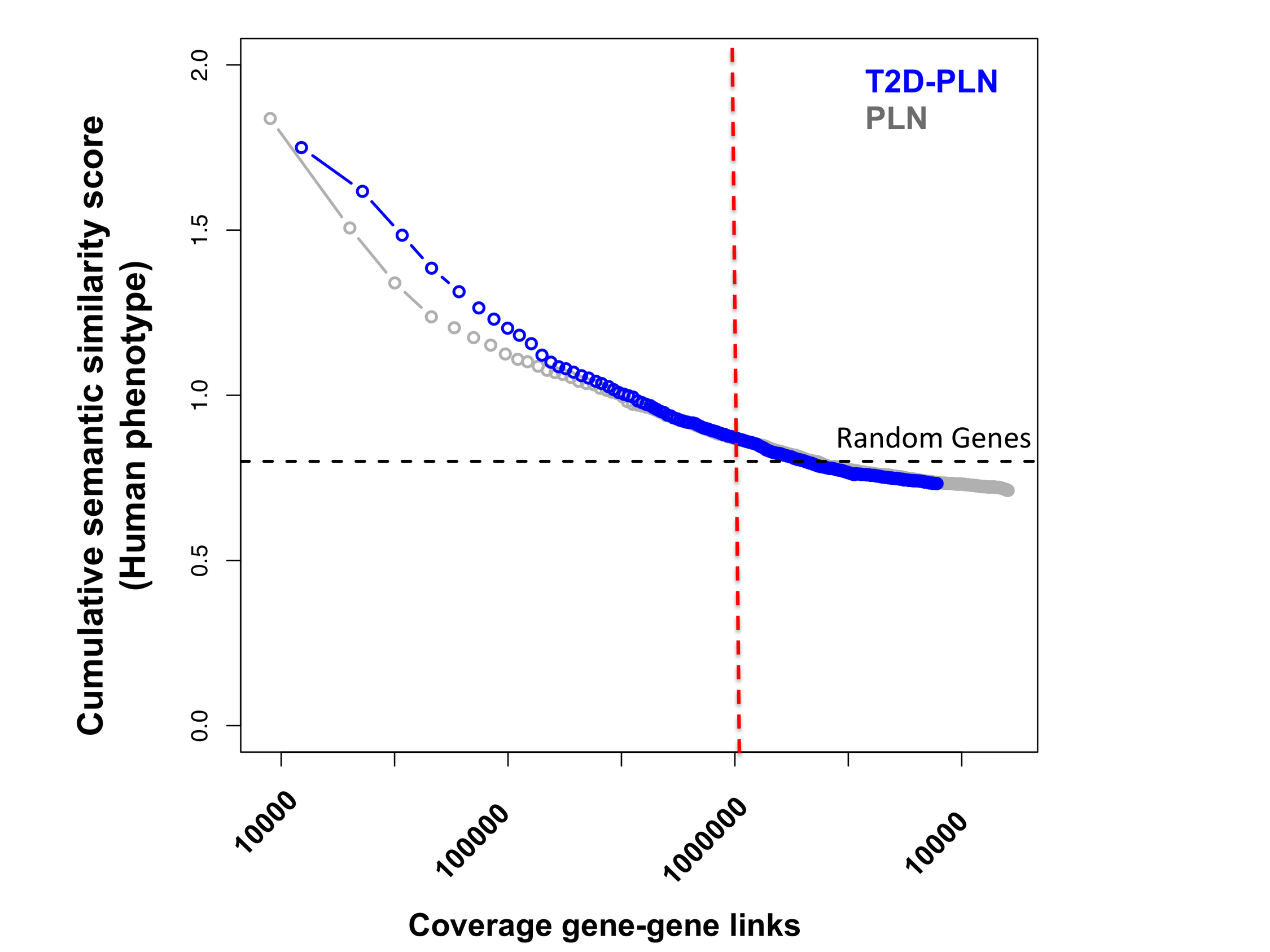

Supplement: S10 Fig — The median of semantic similarity score between gene pairs was determined from their human phenotype annotations database (http://www.human-phenotype-ontology.org/) [53]. Gene pairs were sorted according to the weight of their link either within the un-specific PLN (black curve) or the T2D-PLN (blue curve). One point represents a bin of 500 gene pairs. (TIF) [file pcbi.1005816.s010.tif]

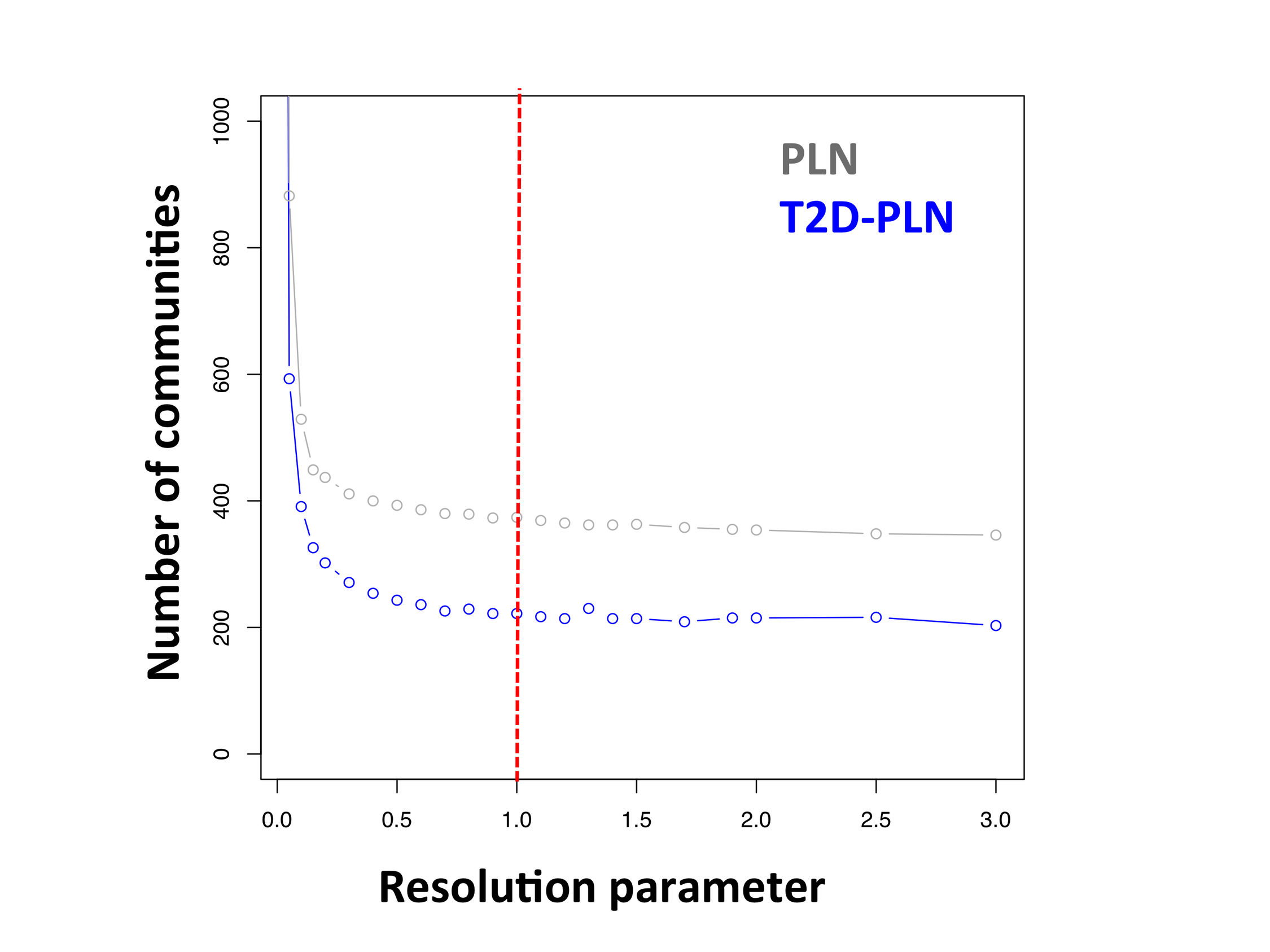

Supplement: S11 Fig — The gray and blue curves represent the number of communities formed when clustering the un-specific PLN and T2D-PLN network, respectively. (TIF) [file pcbi.1005816.s011.tif]

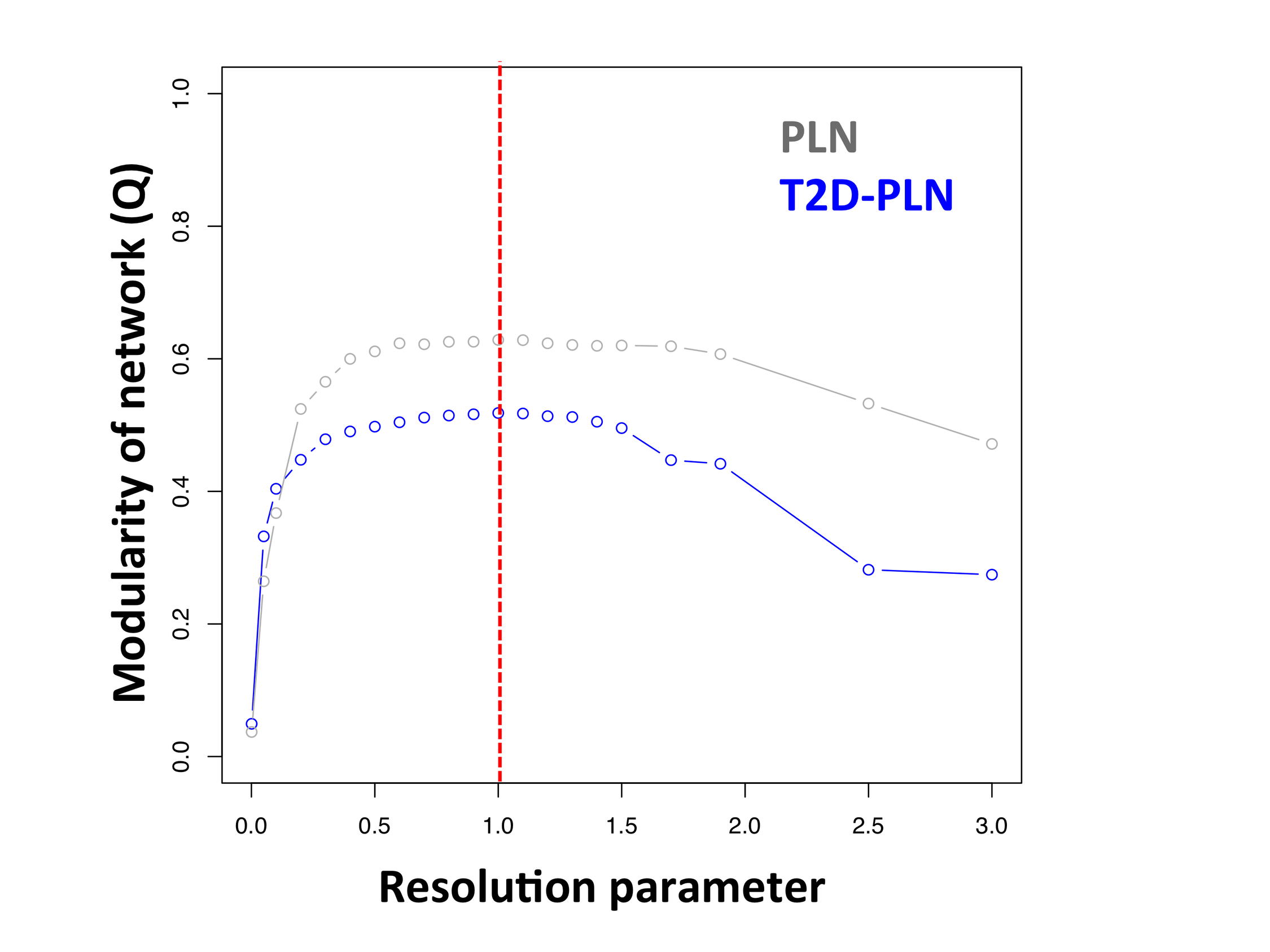

Supplement: S12 Fig — (TIF) [file pcbi.1005816.s012.tif]

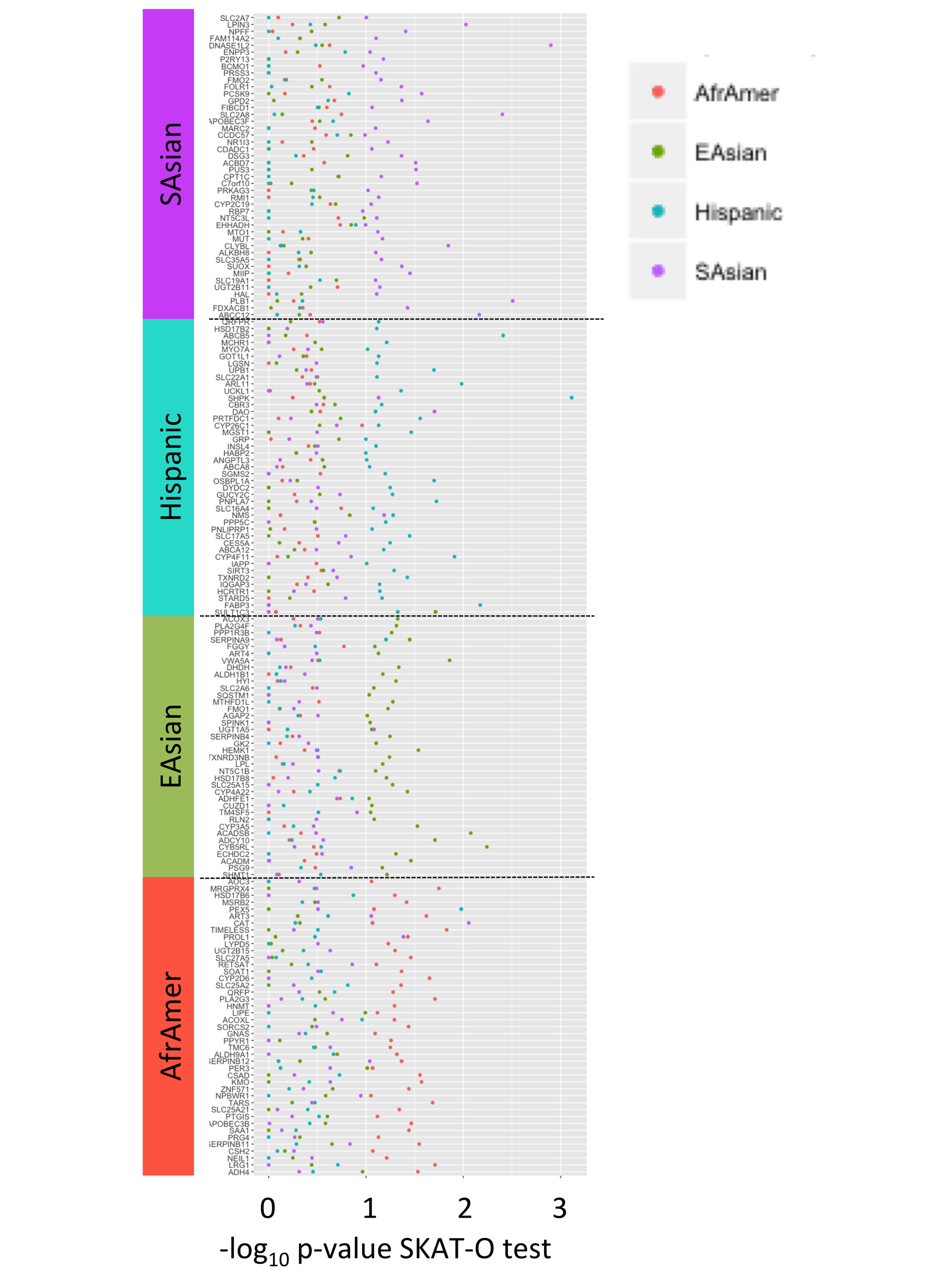

Supplement: S13 Fig — For 40, 42, 51, 45 Community 5 genes impacted by T2D-risk PT-variants in the African-American, Hispanic, East-Asian, South-Asian samples, respectively (rows), we report the–log10 of p-value associations from the SKAT-O tests for these genes within each of four ethnic samples (four points per row, following the Y-axis ethnic sample colourings.). (TIF) [file pcbi.1005816.s013.tif]
